# Nuclear RNA surveillance complexes silence HIV-1 transcription

**DOI:** 10.1371/journal.ppat.1006950

**Published:** 2018-03-19

**Authors:** Xavier Contreras, Kader Salifou, Gabriel Sanchez, Marion Helsmoortel, Emmanuelle Beyne, Lisa Bluy, Stéphane Pelletier, Emilie Rousset, Sylvie Rouquier, Rosemary Kiernan

**Affiliations:** Institut de Génétique Humaine, CNRS-University of Montpellier UMR9002, Gene Regulation Laboratory, 141 rue de la cardonille, Montpellier, France; Duke University Medical Center, UNITED STATES

## Abstract

Expression from the HIV-1 LTR can be repressed in a small population of cells, which contributes to the latent reservoir. The factors mediating this repression have not been clearly elucidated. We have identified a network of nuclear RNA surveillance factors that act as effectors of HIV-1 silencing. RRP6, MTR4, ZCCHC8 and ZFC3H1 physically associate with the HIV-1 TAR region and repress transcriptional output and recruitment of RNAPII to the LTR. Knock-down of these factors in J-Lat cells increased the number of GFP-positive cells, with a concomitant increase in histone marks associated with transcriptional activation. Loss of these factors increased HIV-1 expression from infected PBMCs and led to reactivation of HIV-1 from latently infected PBMCs. These findings identify a network of novel transcriptional repressors that control HIV-1 expression and which could open new avenues for therapeutic intervention.

## Author summary

Following integration into the host genome, HIV-1 expression is silenced in a small population of cells, largely via epigenetic mechanisms that repress LTR-mediated transcription. This repression creates a reservoir of cells that prevent an effective cure. It is unclear how and why integrated HIV-1 becomes transcriptionally silenced. Here, we identify a network of nuclear RNA surveillance factors that repress HIV transcription and whose loss increases virus expression in latently infected J-Lat and PBMCs. These findings advance the understanding of transcriptional repression of HIV-1.

## Introduction

The human immunodeficiency virus-1 (HIV-1) remains one of the world’s leading health threats with over 36 million people infected worldwide (UNAIDS, Global Report, 2016). Despite significant advances in antiretroviral therapy, it is not currently possible to eradicate HIV-1 infection due to the presence of a small but highly persistent reservoir of infected cells that host transcriptionally repressed proviruses. Transcriptional activation of these proviruses occurs rapidly following cessation of therapy leading to renewed high viral burden. The key to eradicating HIV-1 infection could be found in a more complete understanding of HIV-1 transcription and the mechanisms contributing to transcriptional repression.

Transcription of the HIV-1 proviral DNA is initiated by the binding of RNA polymerase II (RNAPII) to the HIV-1 promoter located in the 5’ long terminal repeat region (LTR). RNAPII recruitment is mediated by viral enhancer and promoter elements. However, HIV-1 transcription is repressed by proximal RNAPII pausing and premature termination [1–3]. Short transcripts accumulate in infected cells in culture as well as in CD4+ T cells from patients [1, 4, 5]. These short transcripts contain a stable stem loop structure known as the trans-activation response region (TAR). Elongation-competent transcription depends on the recruitment of HIV-1 trans-activator protein, Tat which binds the bulge region of TAR RNA, and brings with it the host Super Elongation Complex (SEC) and histone acetyltransferases to suppress RNAPII pausing and premature termination and facilitate processive elongation of transcription [6–8].

While Tat-dependent transactivation has been widely studied, the mechanisms underlying premature termination are less well understood. We have shown that the termination factors, SETX and XRN2 are required for premature termination of HIV-1 transcription, which occurs as a consequence of cleavage of nascent TAR-containing RNA by Microprocessor [3]. This study also revealed a role for the 3′ to 5′ exoribonuclease, RRP6, which generates short HIV-1 transcripts that repress HIV-1 transcription. However, the mechanisms by which HIV-1 is repressed remain unclear. Proviral transcription is also controlled by host epigenetic mechanisms. In the absence of Tat, proviral chromatin is marked by repressive chromatin modifications including H3K27Me3 and H3K9Me2/3 [9–13] as well as the recently described repressive mark, H4K20Me1 [14]. However, the mechanisms contributing to HIV-1 transcriptional repression remain relatively poorly understood.

RRP6 is one of the two catalytic subunits of the nuclear RNA exosome, which is responsible for the quality control of transcripts in the nucleus. The 11-subunit exosome is an essential 3’-to-5’ exoribonuclease complex that degrades or processes nearly every class of cellular RNA [15]. The nuclear RNA exosome consists of a 9-subunit non-catalytic core that binds RRP44 (DIS3) and RRP6 subunits to modulate their processive and distributive exoribonuclease activities, respectively. The nuclear exosome is targeted to its substrates through its co-factor, MTR4 that links it to one of at least 2 RNA-binding complexes, the nuclear exosome targeting (NEXT) complex or the polyA tail exosome targeting (PAXT) connection [16–18].

In this study, we identified a network of nuclear RNA surveillance factors including RRP6-associated factors, MTR4, ZCCHC8 and ZFC3H1, that function as effectors of HIV-1 silencing. RRP6, MTR4, ZCCHC8 and ZFC3H1 were found to interact with RNAPII, associate with the HIV-1 TAR region and repress transcriptional output from the HIV-1 LTR. In the absence of these factors, RNAPII became highly recruited to the promoter. In J-Lat cells that harbor transcriptionally silent HIV-1, knock-down of these factors activated HIV-1 expression and led to the acquisition of H3K36Me3 and H4 pan-acetyl marks associated with the activation of transcription. In PBMCs, loss of the factors increased HIV-1 expression, as measured by p24 antigen assay and RT-qPCR, and furthermore led to reactivation of HIV-1 from latently infected cells. Our findings identify RRP6, MTR4, ZFC3H1 and ZCCHC8 as repressors that dampen transcriptional output from the integrated HIV-1 promoter.

## Results

### Identification of HIV-1 transcriptional repressor proteins

**Fig 1:**
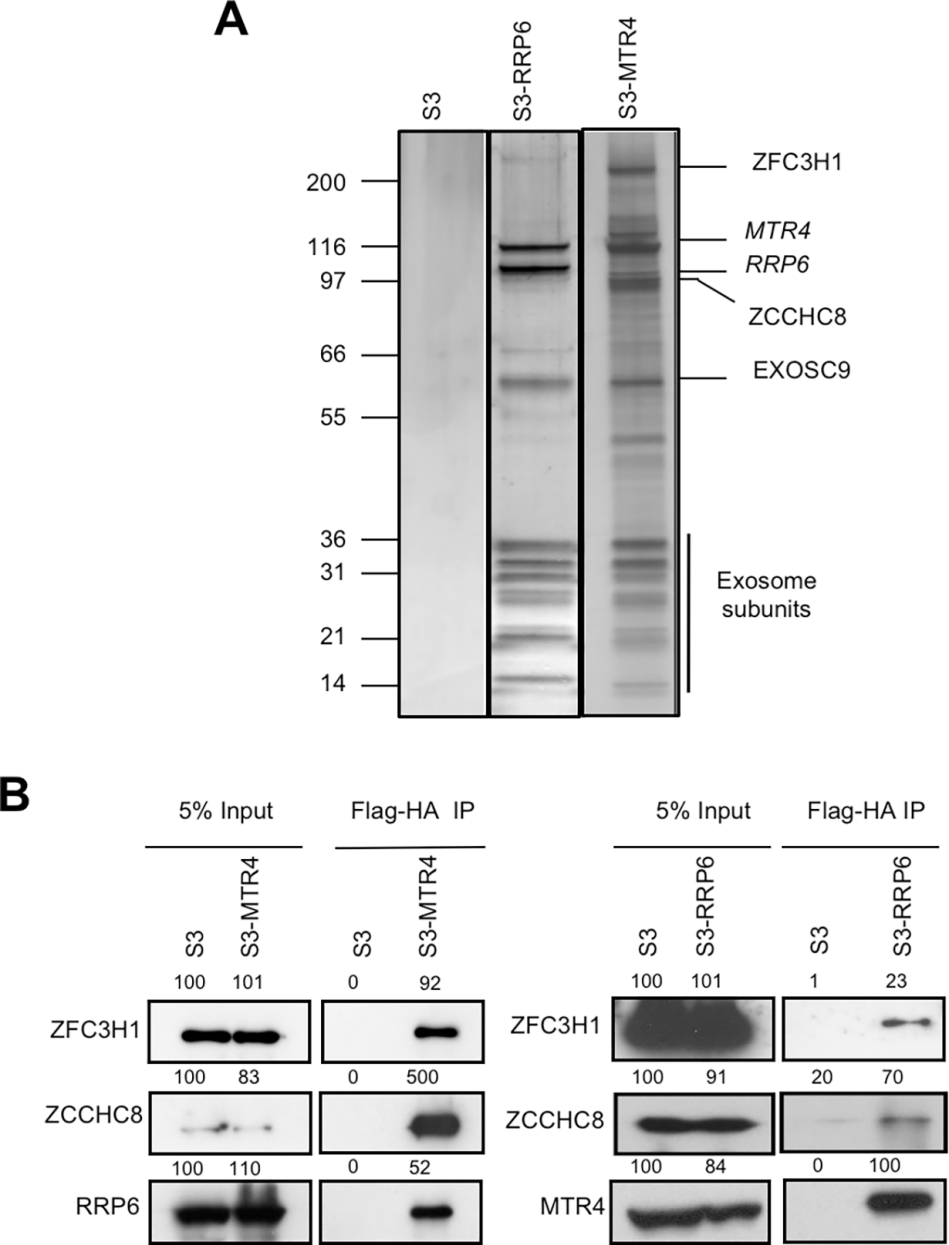
Identification of RRP6 and MTR4 interacting proteins. (A) SDS-PAGE analysis followed by silver staining of eluates of tandem affinity purified Dignam nuclear extracts from HeLa S3 stably expressing Flag-HA-RRP6 or Flag-HA-MTR4 as indicated. (B) Eluates described in A were analyzed by co-immunoprecipitation using the antibodies indicated on the figure. Values shown above the blots represent the intensity of the bands relative to S3 input samples, which were set to 100%. See also S1 Fig.

To investigate mechanisms of HIV-1 transcriptional repression, we identified the interactome of a known HIV-1 repressor, RRP6 [3], and nuclear exosome co-factor, MTR4, by tandem-affinity purification followed by mass spectrometry (Fig 1A and S1 Table). Among the 235 and 202 interactants identified for RRP6 and MTR4, respectively, approximately 50% were found in common (S1A Fig). Among those identified, 81 RRP6 and 71 MTR4 interactants were also identified in a previous study carried out in HEK293 cells [17] (S1B and S1C Fig). These proteins likely represent the core interactants of RRP6 and MTR4. All known subunits of exosome were present, as well as exosome-associated factors, C1D and MPP6. Sub-units of the NEXT complex, ZCCHC8 and RBM7, were found in association with both RRP6 and MTR4, while the zinc-knuckle protein and PAXT subunit ZFC3H1 was better represented in association with MTR4. The more loosely associated catalytic subunit DIS3 was not found among the interactants of either RRP6 or MTR4, possibly due to the high stringency of the Dignam extraction protocol used.

**Fig 2:**
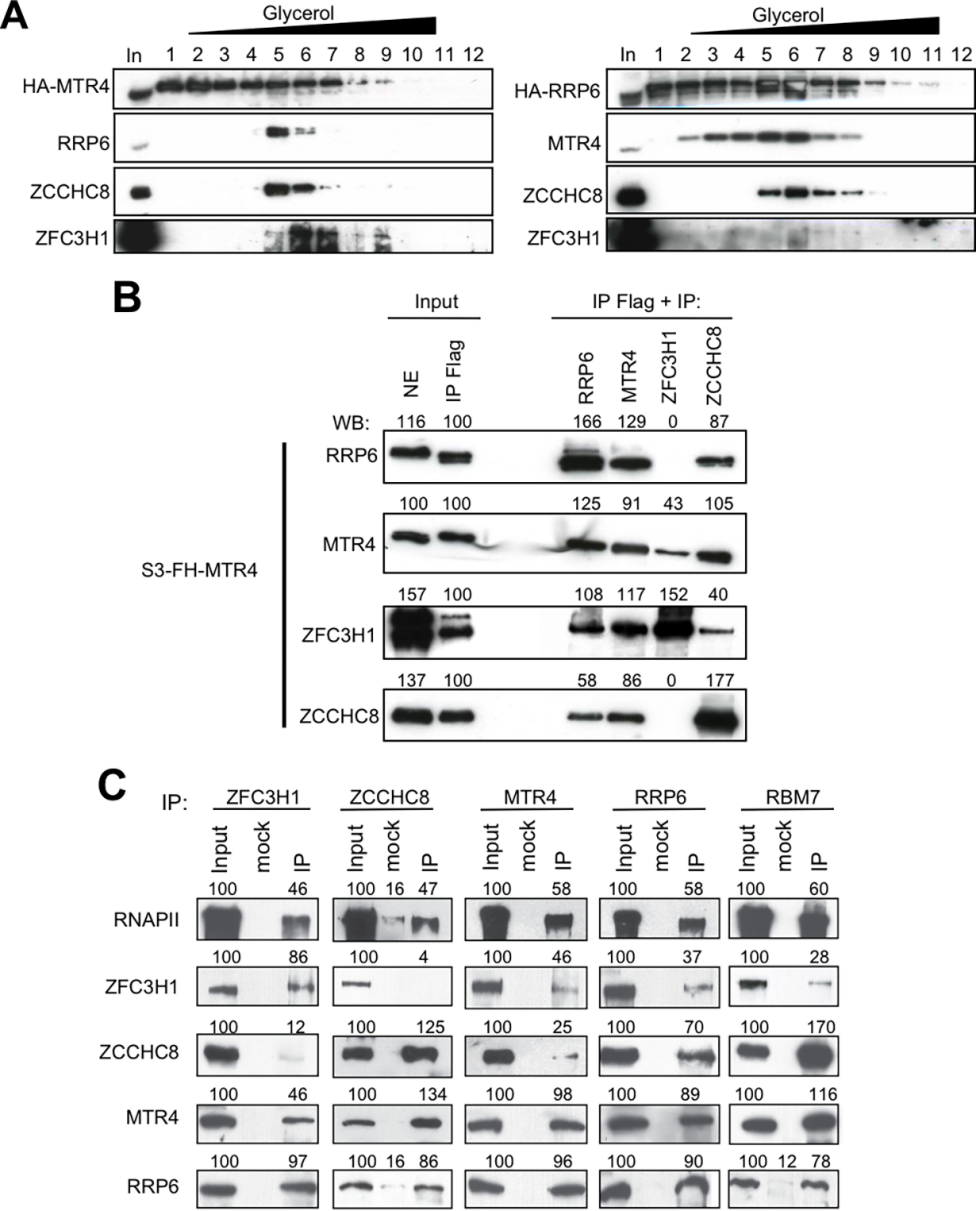
Identification of RRP6- and MTR4- containing complexes. (A) MTR4- or RRP6-containing complexes were separated by gradient sedimentation analysis followed by immunoblotting using the indicated antibodies. (B) Extracts of cells stably expressing Flag-HA-MTR4 were first immunoprecipitated using anti-Flag then re-immunoprecipitated using the antibodies indicated on the figure. Nuclear extract (NE), anti-Flag immunoprecipitate and the Re-IPs were analyzed by Western blotting using the antibodies indicated. Band intensities quantified by ImageJ are shown above the blots. Samples of the first IP (input IP Flag) were set to 100%. (C) Co-immunoprecipitation analysis of ZFC3H1, ZCCHC8, MTR4, RRP6 and RBM7 was performed using the antibodies indicated on the figure. Values shown above the blots represent the intensity of the bands relative to input samples, which were set to 100%. See also S2 Fig and S3 Fig.

Interactions between MTR4, RRP6, ZFC3H1 and ZCCHC8 that were suggested by mass spectrometry were supported by co-immunoprecipitation analysis (Fig 1B). While MTR4 interacted robustly with all factors, association between RRP6 and ZFC3H1 was less robust than with either MTR4 or ZCCHC8, as suggested by mass spectrometry analysis. Glycerol gradient sedimentation analysis of MTR4-associated complexes revealed a complex consisting of at least MTR4, RRP6 and ZCCHC8, which likely corresponds to the NEXT complex (Fig 2A, left panel, fraction 5), and a higher molecular weight complex containing at least MTR4 and ZFC3H1 (Fig 2A, left panel, fraction 7). Similar analysis of RRP6-containing complexes identified the NEXT complex (MTR4, ZCCHC8 and RRP6) (Fig 2A, right panel, fractions 5 and 6) and furthermore confirmed that RRP6-containing complexes are largely devoid of ZFC3H1 (Fig 2A, right panel). These results were confirmed by re-IP analysis (Fig 2B), which showed that MTR4/ZFC3H1 complexes contain little or no RRP6 or ZCCHC8. Co-immunoprecipitation analysis confirmed interactions between the endogenous proteins and furthermore indicated that ZFC3H1 and ZCCHC8 likely occur in distinct complexes (Fig 2C). These results corroborate previous analysis suggesting that MTR4, ZCCHC8 and RBM7 form the NEXT complex [17]. The distinct higher molecular weight MTR4-containing complex is likely to be the recently identified ZFC3H1/MTR4/PABPN1 complex termed PAXT [18].

Interestingly, among the interactants, we found several proteins associated with roles in transcription such as HDAC2, TAF15 and CDK9. Additionally, since several studies including our own point to a direct role of the RNA exosome in transcription and since previous studies showed interaction between RRP6 and RNAPII in Drosophila, we wondered whether human RRP6 and its sub-complexes could interact with RNAPII. Consistent with this idea, we found that subunits of both complexes physically interact with RNAPII (Fig 2C). Furthermore, both ZFC3H1 and ZCCHC8 partially co-localized with RNAPII in the nucleoplasm (S2 Fig).

### Nuclear RNA surveillance complexes repress HIV-1 transcription

**Fig 3:**
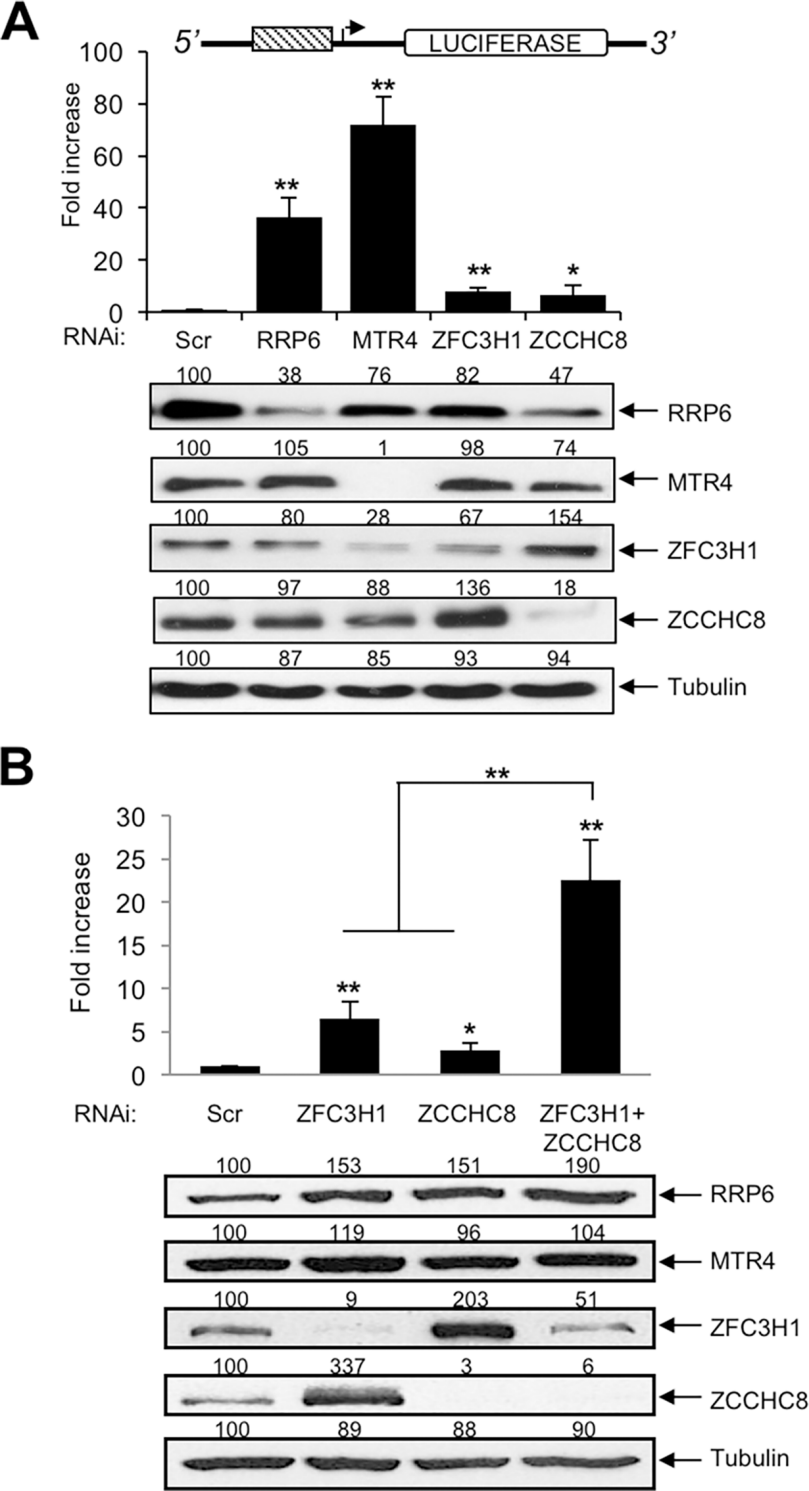
Ablation of RRP6, MTR4 and ZFC3H1/ZCCHC8 stimulates LTR-directed transcript abundance. (A) HeLa-LTR-luc cells were transfected with siRNAs directed against RRP6, MTR4, ZFC3H1, ZCCHC8 or a control siRNA (Scr). Cell extracts were harvested at 60 hr post-transfection and analyzed by luciferase assay and immunoblotting using the antibodies indicated. Values shown above the blots represent the intensity of the bands relative to the control transfection (Scr) samples, which were set to 100%. Fold activation was calculated relative to the control transfection (Scr), which was attributed a value of 1. Data represent mean ± SEM obtained from 3 independent experiments (**P < 0.01, *P < 0.05, independent Student’s *t* test). (B) HeLa-LTR-luc cells were transfected with siRNAs directed against ZFC3H1, ZCCHC8 either alone or in combination, or a control siRNA (Scr). Cell extracts were harvested at 60 hr post-transfection and analyzed by luciferase assay and immunoblotting using the antibodies indicated. Values shown above the blots represent the intensity of the bands relative to the control transfection (Scr) samples, which were set to 100%. Fold activation was calculated relative to the control transfection (Scr), which was attributed a value of 1. Data represent mean ± SEM obtained from 3 independent experiments (**P < 0.01, *P < 0.05, independent Student’s *t* test).

Since we have previously shown that RRP6 is required for processing of TAR RNA that is implicated in transcriptional repression of HIV-1 [3], we wondered whether RRP6-interacting factors might also be implicated in repression of HIV-1. We first performed knock-down of RRP6, MTR4, ZFC3H1 and ZCCHC8 individually and analyzed the effect on LTR-driven luciferase activity (Fig 3A). Knock-down of either RRP6 or MTR4 significantly increased LTR activity, whereas loss of either ZFC3H1 or ZCCHC8 had a very modest effect. Interestingly, immunoblot analysis of the knocked-down cells indicated cross-regulation of the abundance of the different factors (Fig 3A, lower panel). Notably, knockdown of ZFC3H1 enhanced expression of ZCCHC8, and vice versa. Given that others [18] and we show that ZFC3H1 and ZCCHC8 exist in largely distinct complexes, the results shown in Fig 3A suggest that these factors may be partially redundant and their activities may be compensatory. To test this, simultaneous knock-down of ZFC3H1 and ZCCHC8 was performed. As shown in Fig 3B, only the double knock-down successfully diminished the expression of both factors and significantly increased LTR-driven luciferase activity. We also tested the effect of RRP6 co-factor C1D, core exosome components and the second exosome-associated RNase, DIS3. Knock-down of C1D increased luciferase activity (S3A Fig). Conversely, simultaneous knock-down of 3 core exosome subunits (EXOSC2, EXOSC7 and EXOSC9) or of DIS3 had no effect on LTR-driven luciferase activity (S3B Fig). Thus, RNA processing factors RRP6, MTR4, ZFC3H1, ZCCHC8 and C1D are implicated in the repression of LTR-driven activity, in a manner that is independent of core exosome and DIS3.

**Fig 4:**
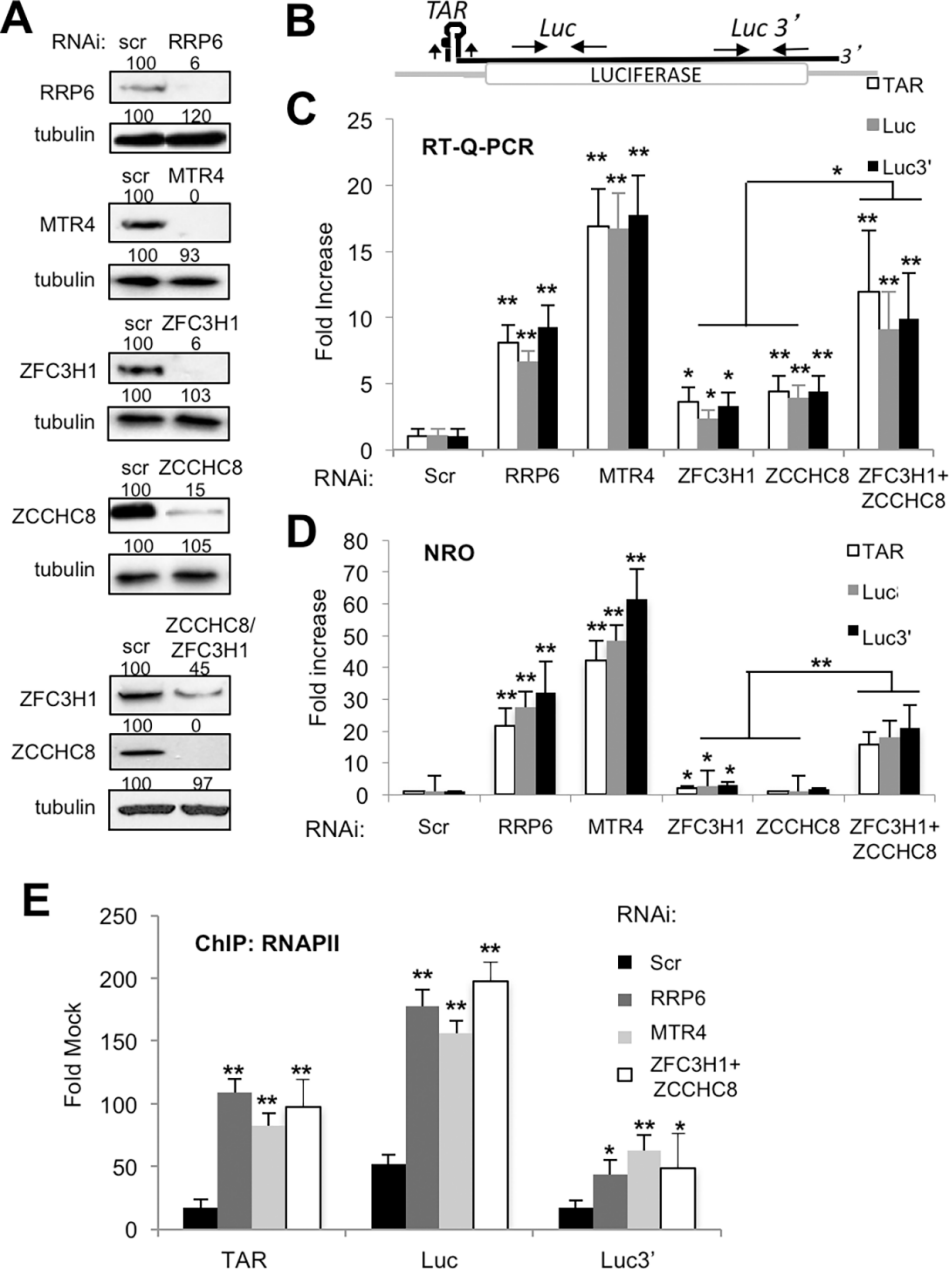
RRP6, MTR4, ZFC3H1/ZCCHC8 silence LTR-driven transcription. (A) HeLa-LTR-luc cells were transfected with siRNAs directed against RRP6, MTR4, ZFC3H1, ZCCHC8, ZFC3H1+ZCCHC8 or a control siRNA (Scr). Cell extracts were harvested at 60 hr and analyzed by immunoblotting using the antibodies indicated. Values shown above the blots represent the intensity of the bands relative to the control transfection (Scr) samples, which were set to 100%. (B) Schematic diagram showing the locations of primers used to amplify sequences by Q-PCR. The *luciferase* reporter gene is shown as a white box. TAR RNA stem-loop is indicated at the 5’ end of the transcript. Primers used to amplify TAR and coding region sequences are indicated on the figure. (C) Total RNA isolated from cells described in A was analyzed by RT-q-PCR using the oligonucleotide pairs indicated, and GAPDH. The amount of the indicated mRNA was normalized to the amount of GAPDH mRNA in each sample, and the values were normalized to those for the control transfection (Scr), which was attributed a value of 1. Data represent mean ± SEM obtained from 3 independent experiments (**P < 0.01, *P < 0.05, independent Student’s *t* test). (D) HeLa-LTR-luc cells transfected with the indicated siRNAs were analyzed by nuclear run-on transcription. RT-q-PCR was performed using the indicated primer pairs. Results are shown relative to a control sample (scr), which was attributed a value of 1. Data represent mean ± SEM obtained from 3 independent experiments (**P < 0.01, *P < 0.05, independent Student’s *t* test). (E) HeLa-LTR-luc cells transfected with the indicated siRNAs were analyzed by chromatin immunoprecipitation for RNAPII. PCR was performed using the indicated primer pairs. Results are shown relative to an IgG control. Data represent mean ± SEM obtained from 3 independent experiments (**P < 0.01, *P < 0.05, independent Student’s *t* test).

We next analyzed the effect of knock-down of each factor on LTR-directed mRNA synthesis. As shown in Fig 4A–4C, loss of RRP6, MTR4 or double knock-down of ZFC3H1 and ZCCHC8 increased the abundance of LTR-directed mRNA. Given that the factors are involved in RNA degradation in the nucleus, we sought to determine whether the increase in LTR activity observed following loss of these factors was due to a direct effect on HIV-1 transcription or rather through stabilization of HIV-1 RNA. We performed nuclear run-on transcription (NRO) analysis that specifically measures nascent transcripts to distinguish an effect on transcription from that of RNA stabilization. Fig 4D shows that RRP6, MTR4 and ZFC3H1/ZCCHC8 increased LTR-driven nascent transcript synthesis. To confirm that these factors indeed modulate LTR-driven transcription, chromatin immunoprecipitation (ChIP) of RNAPII was performed in control or knock-down conditions. Significant recruitment of RNAPII was observed following loss of RRP6, MTR4 or ZFC3H1/ZCCHC8 (Fig 4E), reflecting results obtained by luciferase assay, RT-qPCR and nuclear run-on transcription (Figs 3 and 4A–4D). We noted that loss of RRP6, MTR4 or ZFC3H1/ZCCHC8 had no significant impact on the expression of several host factors that modulate HIV-1 transcription, including SupT6H, PAF1, AF9, HDAC1 or RNAPII (S4 Fig). Taken together, these findings suggest that RRP6, MTR4 and exosome targeting factors ZFC3H1 and ZCCH8 co-operate to control HIV-1 LTR-driven transcriptional activity.

**Fig 5:**
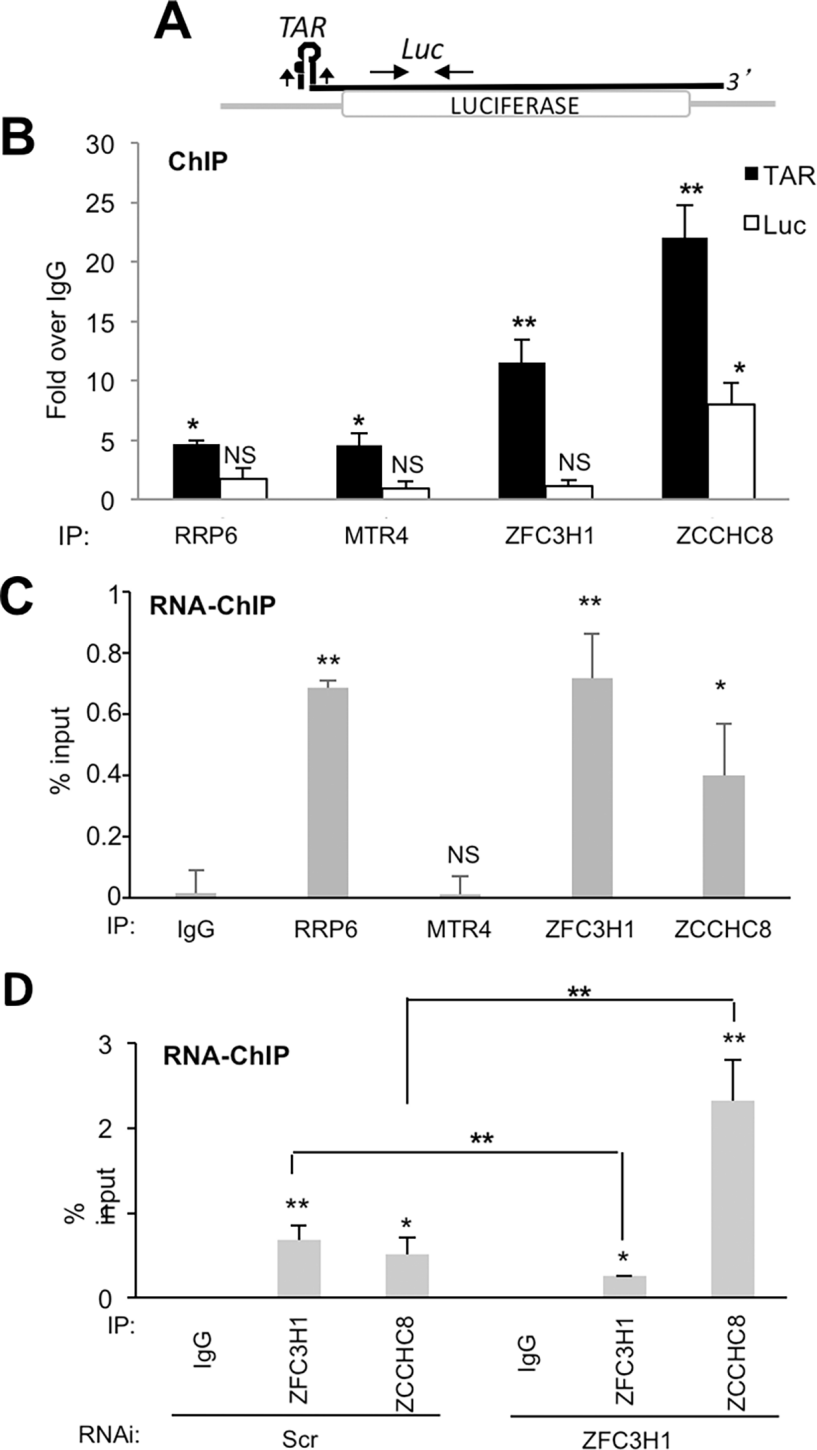
RRP6, MTR4, ZFC3H1 and ZCCHC8 associate with the promoter-proximal region of HIV-1 chromatin. (A) Schematic diagram showing the location of primers used to amplify ChIP samples by Q-PCR. Primers used to amplify promoter-proximal and coding region sequences are indicated on the figure. (B) HeLa-LTR-luc cells were analyzed by ChIP for association of RRP6, MTR4, ZFC3H1 and ZCCHC8 with the promoter-proximal and coding regions of LTR-luc, Results are shown relative to an IgG control. Data represent mean ± SEM obtained from 3 independent experiments (**P < 0.01, *P < 0.05, NS indicates not significant, independent Student’s *t* test). (C) HeLa-LTR-luc cells were analyzed by RNA-ChIP for association of RRP6, MTR4, ZFC3H1 and ZCCHC8 with the promoter-proximal region of LTR-luc. Data represent mean ± SEM obtained from 3 independent experiments (**P < 0.01, *P < 0.05, NS indicates not significant, independent Student’s *t* test). (D) HeLa-LTR-luc cells transfected with the indicated siRNAs were analyzed by RNA-ChIP for association of ZFC3H1 and ZCCHC8 with the promoter-proximal region of LTR-luc. Data represent mean ± SEM obtained from 3 independent experiments (**P < 0.01, *P < 0.05, NS indicates not significant, independent Student’s *t* test).

To determine whether, like RRP6, MTR4, ZFC3H1 and ZCCHC8 are directly implicated in the repression of HIV-1 transcription, we performed ChIP analysis since we would expect to find repressive factors associated with HIV-1 chromatin. ChIP analysis revealed that RRP6, MTR4, ZFC3H1 and ZCCHC8 are associated with HIV-1 chromatin, particularly with the transcription start site, TAR (Fig 5A and 5B). We next performed RNA-ChIP analysis, which measures chromatin association of factors implicated in co-transcriptional RNA processing [19]. This analysis suggested that RRP6, ZFC3H1 and ZCCHC8 are in close contact with TAR RNA, while MTR4 may be less stably associated with HIV-1 RNA molecules (Fig 5C). Indeed, ZFC3H1 and ZCCHC8 belong to complexes that target nuclear exosome to its RNA substrates [16–18]. Since ZCCHC8 and ZFC3H1 showed cross-regulation of expression, we analyzed the association of these factors following loss of ZFC3H1. Interestingly, loss of ZFC3H1 led to enhanced recruitment of ZCCHC8 consistent with the idea that ZCCHC8 functionally compensates for the loss of ZFC3H1 (Fig 5D).

### Nuclear surveillance factors are associated with transcriptionally repressed HIV-1

**Fig 6:**
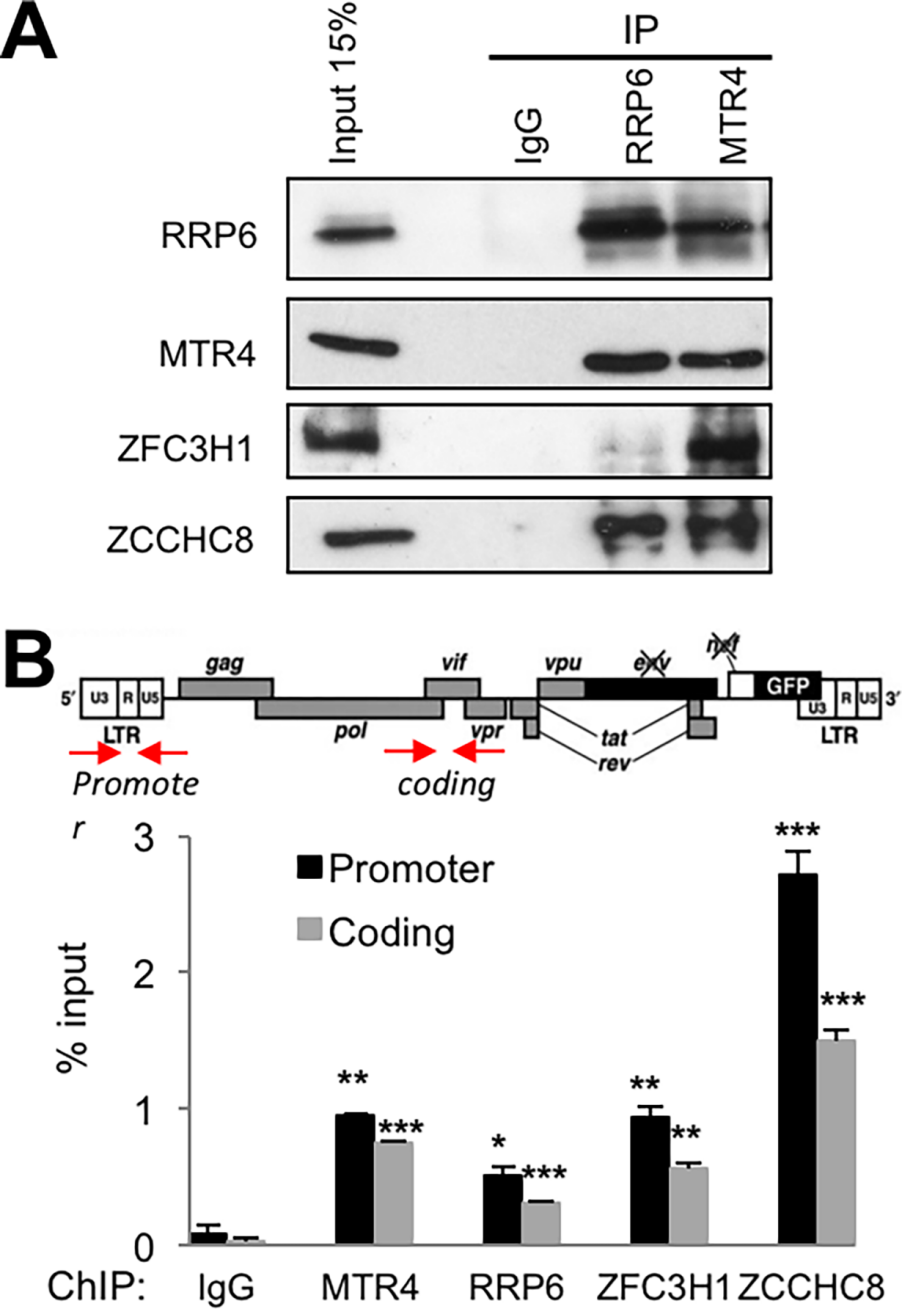
RRP6, MTR4, ZFC3H1 and ZCCHC8 interact and are associated with HIV-1 chromatin in J-Lat cells. (A) Nuclear extracts of J-Lat (clone 10.6) cells treated with a combination of 20 ng/ml TNFα and 20 mM TSA were used for co-immunoprecipitation analysis of MTR4 and RRP6 using the antibodies indicated on the figure. (B) J-Lat (clone 10.6) cells were analyzed by ChIP for association of RRP6, MTR4, ZFC3H1 and ZCCHC8 or an IgG control with the promoter and coding regions of HIV-1. A schematic diagram showing the locations of primers used to amplify sequences present in chromatin immunoprecipitates by Q-PCR is shown above the graph. Data represent mean ± SEM obtained from 3 independent experiments (***P < 0.001, **P < 0.01, *P < 0.05, independent Student’s *t* test).

We next wished to determine whether nuclear surveillance factors may affect expression of HIV-1 in the context of a full viral genome. We first analyzed the interactions between RRP6, MTR4, ZFC3H1 and ZCCHC8 in the presence of HIV-1 proteins. In order to do so, we used J-lat 10.6 cell line which harbors a repressed HIV genome in which Nef has been replaced by GFP [20]. To induce viral protein expression and HIV-1 particle production, the cells were activated with a combination of 20 ng/ml TNFα and 20 nM trichostatin A (TSA). Under these conditions, 80% of cells were positive by flow cytometry and p24 was present in the cell culture media (34 ng/ml), confirming the production of HIV-1 proteins. Co-immunoprecipitation analysis showed that, similarly to that observed in HeLa cells, MTR4 interacted with RRP6, ZFC3H1 and ZCCHC8 while RRP6 interacted with MTR4 and ZCCHC8 and modestly with ZFC3H1 in the presence of HIV-1 proteins (Fig 6A).

We next determined whether the repressive factors are associated with HIV-1 chromatin in J-Lat cells. ChIP analysis revealed that MTR4, RRP6, ZFC3H1 and ZCCHC8 are associated with HIV-1 DNA in J-Lat cells (Fig 6B). Thus, nuclear surveillance factors appear to form molecular complexes in HIV-1 target cells in the presence of viral proteins, and to be associated with HIV-1 chromatin.

### Loss of nuclear RNA surveillance complexes activates HIV-1 expression

**Fig 7:**
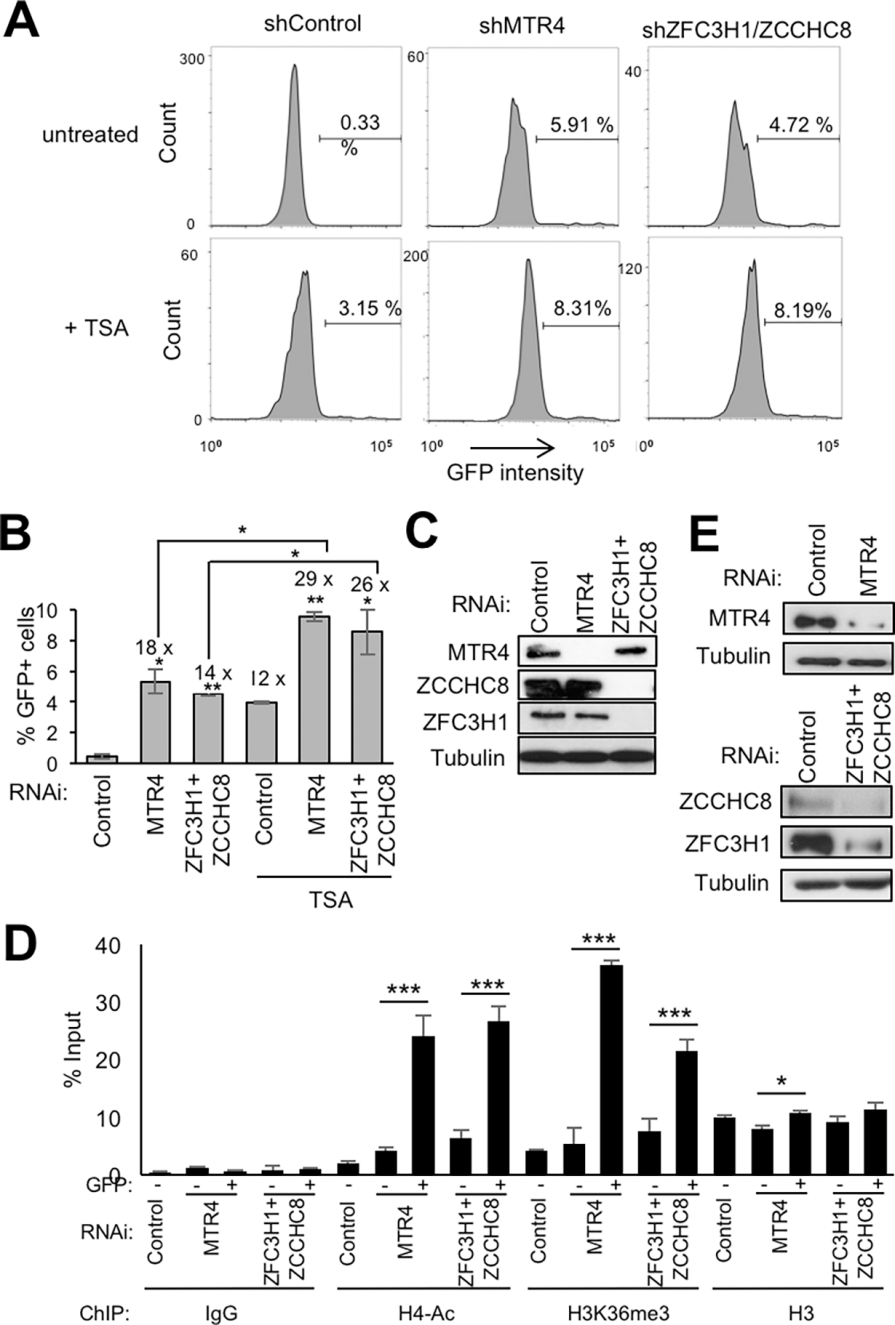
Nuclear surveillance factors modulate HIV expression in J-Lat cells. (A-C) J-Lat (clone 10.6) cells infected with the indicated shRNAs for 7 days, treated or not with 100 nM TSA for 24 hr, were analyzed by flow cytometry for GFP expression. (A) Representative profiles are shown. (B) Graphs represent mean ± SEM of GFP-positive cells obtained from 4 independent experiments (**P < 0.01, *P < 0.05, independent Student’s *t* test). Numbers indicated above the columns represent fold increase over the untreated control. (C) An aliquot of cells was analyzed by direct western blotting using the antibodies indicated. (D-E) J-Lat (clone 10.6) cells were transduced with lentiviral particles expressing the indicated shRNAs. Cells expressing GFP or not were separated by cell sorting 7 days later. (D) ChIP analysis was performed using the indicated antibodies on GFP expressing (+) and non-expressing (-) cells. Graphs represent mean ± SEM from 3 independent experiments (***P < 0.001, * *P* < 0.05, independent Student’s *t* test). (E) An aliquot of cells harvested prior to sorting was analyzed by direct western blotting using the antibodies indicated.

We next wondered whether the repressive factors might be involved in the control of HIV-1 virus expression. Thus, J-Lat 10.6 cells were transduced with shRNAs targeting either MTR4 or ZFC3H1 and ZCCHC8 (Fig 7A). Expression of HIV-1 was detected by flow cytometry (Fig 7B and 7C). Knock-down of either MTR4 or ZFC3H1+ZCCHC8 activated HIV-1 expression to a level similar to that observed following treatment with the HDAC inhibitor, TSA. Loss of MTR4 or ZFC3H1+ZCCHC8 appeared to have an additive effect with TSA, resulting in expression of HIV-1 in nearly 10% of cells.

We next investigated whether the activation of HIV-1 expression following loss of nuclear surveillance factors was accompanied by changes to HIV-1 chromatin. Thus, chromatin modifications associated with the activation of transcription, H3K36Me3 and H4 acetylation, were analyzed in cells expressing HIV-1 following loss of either MTR4 or ZFC3H1 +ZCCHC8. ChIP analysis showed the presence of histone marks associated with the activation of transcription were significantly increased while association of histones, as measured by histone H3, remained mostly unaffected (Fig 7D). These results suggest that MTR4, ZFC3H1/ZCCHC8 are implicated in the regulation of HIV-1 expression in J-Lat cells.

**Fig 8:**
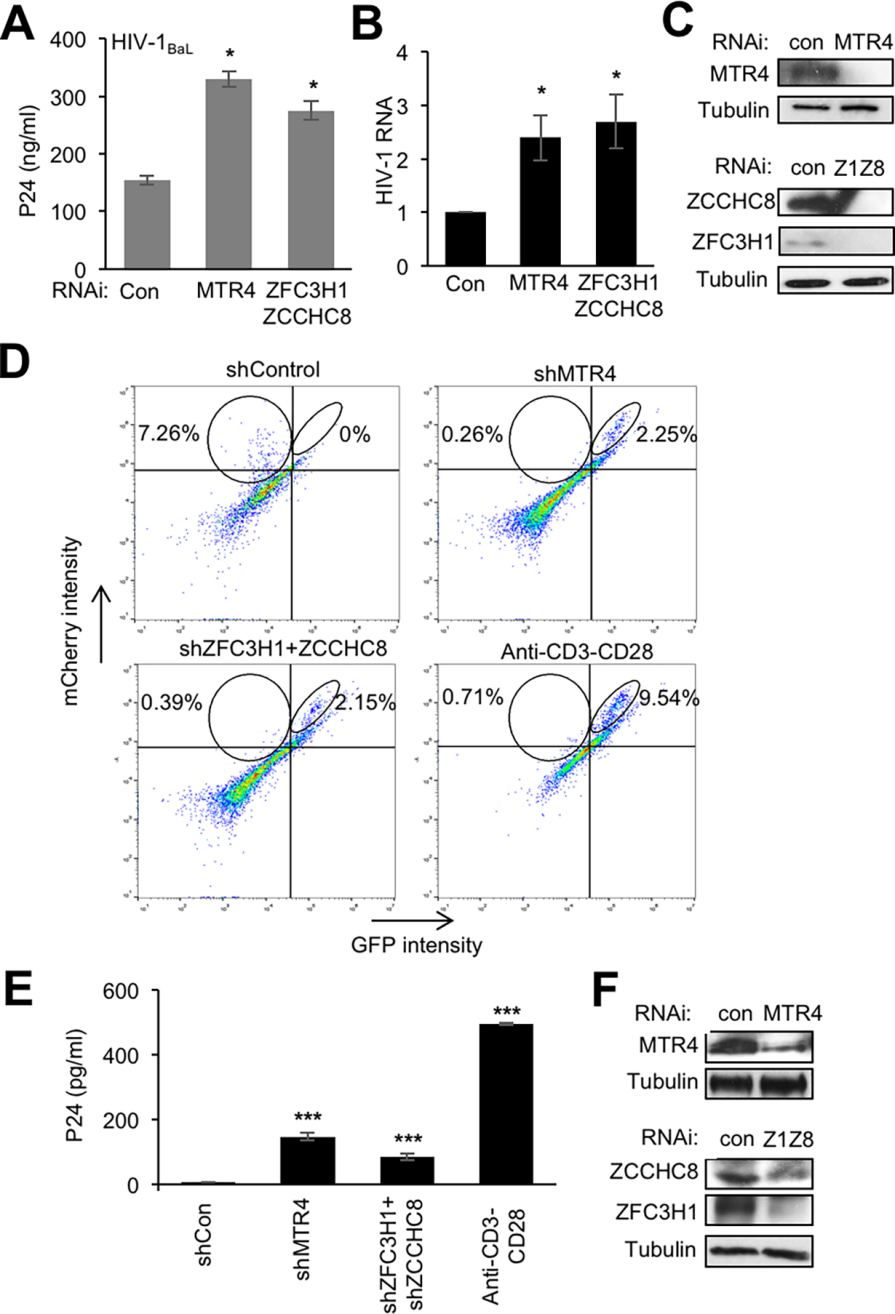
Nuclear exosome factors modulate HIV-1 expression in PBMCs. (A-C) PBMCs from healthy donors were activated using anti-CD3/CD28 beads and IL2 (30 U/ml) for 3 days then infected with HIV-1_BaL_. Cells were transduced with lentiviral particles expressing the indicated shRNAs 3 days post-infection. Cell extracts and culture supernatants were harvested 7 days later. (A) Graph represents mean ± SEM of p24 concentration in cell culture supernatants obtained from 3 experiments performed on different donor cells (*P < 0.05, independent Student’s *t* test). (B) RT-qPCR analysis of cell extracts from (A) using primers amplifying the Vif region (*P < 0.05, independent Student’s *t* test). (C) An aliquot of cells was analyzed by direct western blotting using the antibodies indicated. (D-F) Activated CD4+ T-cells from healthy donors were infected with HIV-1_DuoFluo_ and latently infected cells isolated by cell sorting were transduced with lentiviral particles expressing the indicated shRNAs. Cells were analyzed by flow cytometry and p24 ELISA 7 days later. (D) Representative profiles are shown. Circled areas on each profile show mCherry single positive cells (left circle) and mCherry and GFP double positive cells (right circle). Numbers indicate the percentage of cells of the total within the circles. (E) Quantification of p24 present in culture supernatants. Graphs represent mean ± SEM obtained from 3 experiments using PBMC from different donors (***P < 0.001, independent Student’s *t* test). (F) An aliquot of cells was analyzed by direct western blotting using the antibodies indicated. See also S5 Fig.

We next wanted to determine whether nuclear surveillance factors are also relevant for the control of HIV-1 expression in peripheral blood mononuclear cells (PBMCs). Thus, CD4^+^ T cells were purified from PBMCs, activated using α-CD3/CD28 and IL2 then infected with HIV-1_BaL_ as previously described [21]. At 3 days post-infection, cells were transduced with lentiviral particles expressing shRNAs targeting MTR4, ZFC3H1+ZCCHC8 or a negative control shRNA. Viral release in the supernatant was quantified 7 days later by p24 ELISA and HIV RNA in cells was measured by RT-qPCR of *vif* sequence. Loss of either MTR4 or ZFC3H1+ZCCHC8 led to an approximately 2-fold increase in HIV-1 particle production and viral RNA compared to a control shRNA (Fig 8A–8C). These results indicate that nuclear surveillance factors can modulate the expression of HIV-1 in activated PBMCs.

Next, to determine whether the same factors might be implicated in controlling the latent infection of HIV-1, we exploited a PBMC model of latency using HIV-1_DuoFluo_ virus that has been described previously [22]. Briefly, HIV_DuoFluo_ is a full-length HIV-1 genome with GFP in place of Nef and mCherry under the control of an internal EF1α promoter. Productively infected cells that are GFP and mCherry double positive can therefore be distinguished by flow cytometry from latently infected cells, that are mCherry single positive. Thus, α-CD3/CD28 + IL-2-activated CD4+ T-cells were infected with HIV-1_DuoFluo_. Latently infected mCherry positive cells were isolated by FACS sorting and transduced with lentiviruses expressing shRNAs targeting MTR4, ZFC3H1+ZCCHC8 or a control. Ten days later, latent and reactivated cell populations were quantified by flow cytometry (Fig 8D–8F). Although the mCherry signal had diminished in the latently infected cells, possibly due to further silencing of provirus, as reported previously [22], the control sample (shControl) still contained mCherry-expressing single positive cells (7.26%) but did not contain mCherry/GFP-expressing double positive cells (Fig 8D). Importantly, Loss of MTR4 or ZFC3H1/ZCCHC8 led to an increase in the number of double positive cells (approximately 2%) while α-CD3-CD28 activation, used as a positive control, contained 9.5% double positive cells while the population of mCherry single positive cells was diminished in shRNA-treated and α-CD3-CD28-stimulated samples (Fig 8D). Of note, transduction of uninfected PBMCs with shRNAs or stimulation with α-CD3-CD28 did not induce mCherry- or GFP-positive signals (S5 Fig). To confirm that loss of MTR4 or ZFC3H1/ZCCHC8 led to reactivation of latent virus, p24 was measured in the cell culture supernatants concomitantly with FACS analysis (Fig 8E). The shCon sample did not express significant levels of p24, suggesting that these cells were latently infected, while a modest increase in p24 was detectable in shMTR4 and shZFC3H1/ZCCHC8 samples relative to the α-CD3-CD28-stimulated positive control sample. These findings suggest that MTR4, ZFC3H1 and ZCCHC8 are implicated in the silencing of HIV-1 virus.

## Discussion

Following integration, the HIV-1 LTR is subjected to transcriptional repression in a small population of cells that contribute to establishment of the latent reservoir. The mechanisms that mediate repression of the LTR are poorly understood. In this study, we identify a network of nuclear exosome factors that potently represses LTR-directed transcription and downregulates HIV-1 expression in J-lat cells and PBMCs.

We have previously shown that exosome-associated distributive 3’ to 5’ RNase, RRP6 is implicated in transcriptional repression of the LTR through a mechanism that requires processing of TAR RNA [3]. In the present study, we show that MTR4, C1D, ZCCHC8 and ZFC3H1 are also required for repression. Interestingly, repression does not appear to depend on core exosome subunits or the exosome-associated processive RNase activity of DIS3. Recent studies have described the crystal structure of the 11-subunit nuclear exosome [23, 24]. The barrel-shaped 9-subunit core associates with the processive exo- and endoribonuclease DIS3 at the bottom and the distributive exoribonuclease RRP6 at the cap. These structures have identified several possible paths for RNA following its engagement by the exosome that explain the substrate specificities of RRP6, DIS3 and core exosome. Among exosome subunits, LTR repression depends on RRP6, MTR4 and C1D. In contrast, core exosome and DIS3 appear to be dispensable, suggesting that the processed RNA probably does not pass through the central core exosome channel leading to DIS3. Indeed, an alternative route in which the RNA substrate does not pass through the central channel but directly reaches the active center of RRP6 has recently been described [23]. Furthermore, this could suggest that the substrate contains less than 30 nt of unstructured RNA, which is the length spanned by the central channel, and/or possesses a 3’ OH that can be processed by RRP6 but not DIS3. Interestingly, 3’ OH ends are generated by cleavage reactions, including endonucleolytic cleavage mediated by Microprocessor.

The nuclear exosome is recruited to its substrates by MTR4-containing complexes such as NEXT, comprised of ZCCHC8 and RBM7, or PAXT, comprised of ZFC3H1 and PABPN1, respectively. Although NEXT or PAXT target distinct subtypes of cellular RNAs, some RNAs are targeted by both complexes [18]. Indeed, our data suggest that both complexes contribute to repression of HIV-1 transcription. Depletion of both complexes was required to de-repress LTR transcription. This could suggest that recruitment of nuclear exosome by either pathway is sufficient for HIV-1 transcriptional repression.

It remains unclear precisely how recruitment of nuclear exosome represses transcription of the HIV-1 LTR. None of the identified factors is known to interact with or recruit repressive complexes, such as H3K9 or H3K27 methyltransferases. R-loops formed over pause-site termination regions lead to the recruitment of G9A and formation of H3K9Me2 repressive mark, which in turn reinforces RNAPII pausing and promotes termination of transcription at human genes [25]. Since we have previously established a role for RRP6, together with Microprocessor, SETX and XRN2 in premature termination of HIV-1 transcription [3], it is possible that the newly identified repressors of HIV-1 act together with RRP6 to mediate or maintain facultative heterochromatin near the LTR to provoke premature termination of HIV-1 transcription. Alternatively, the terminated HIV-1 transcripts may provide a platform for H3K9Me2/3 formation to reinforce transcriptional repression. Further studies will be required to determine the role of RRP6, MTR4, ZFC3H1 and ZCCHC8 in mediating or maintaining transcriptional repression of HIV-1. The extent to which nuclear surveillance factors can also repress host genes is currently not known. However, we noted that expression of some key factors for HIV-1 transcription, such as Supt6H, PAF1, SEC subunit AF9, HDAC1, or RNAPII itself, was not affected by loss of nuclear exosome.

Nuclear exosome has recently been identified as a necessary co-factor in the life cycle of another RNA virus, Influenza A virus (IAV) [26]. By interacting with nuclear exosome, the viral polymerase is recruited to the vicinity of actively degraded cell transcripts that are used as a source for ‘cap snatching’, a pre-requisite for the synthesis of IAV transcripts. Thus, IAV has co-opted an essential cellular process, RNA degradation by nuclear exosome, for the benefit of its own life cycle. In the context of HIV-1 infection, one might consider that a long latency phase is beneficial for the pathogenicity of the virus and that nuclear exosome, along with other cellular machineries, have been co-opted for this purpose.

These findings provide new insights into the mechanisms involved in transcriptional repression of HIV-1. The factors identified, RRP6, MTR4, ZFC3H1 and ZCCHC8, are all associated with the nuclear exosome and required for nuclear RNA surveillance of the host cell. Interestingly, subunits of the core exosome, or the second 3’ to 5’ RNase activity associated with nuclear exosome, DIS3, had no effect on HIV-1 transcription. Further studies will be required to unravel the mechanism by which these factors, which are physically associated with HIV-1 chromatin, contribute to its transcriptional repression. This may open new avenues for the development of HIV-1 specific therapies if the interaction between nuclear exosome factors and the HIV-1 LTR could be specifically inhibited.

### Experimental procedures

Details on antibodies, siRNAs and PCR primers used in this study can be found in Supplemental experimental procedures.

### Antibodies and plasmids

Antibodies used in this study are listed in S2 Table. Plasmids encoding pOZ-Flag-HA-RRP6 and pOZ-Flag-HA-MTR4 were cloned using pOZ-N-FH plasmid [27]. Plasmids expressing shRNAs targeting RRP6, MTR4, ZFC3H1 or ZCCHC8 were purchased from Sigma. A plasmid expressing a control shRNA was obtained through Addgene (plasmid 1864). Sequences of shRNAs are shown in S4 Table.

### Cell culture, treatment, transfection and infection

HeLa cells (ATCC) containing a stably integrated LTR linked to a luciferase reporter gene (HeLa LTR-luc cells [28]) were propagated in Dulbecco’s modified Eagle’s medium (DMEM) supplemented with 10% FBS and antibiotics. HEK293T (ATCC) were grown in DMEM with Hepes (25 mM), supplemented with 10% FBS and antibiotics. All cells were grown in a humidified incubator at 37°C with 5% CO2.

HeLa LTR-luc were transfected with siRNAs (30 nM final concentration) using Interferin (PolyPlus Transfection) according to the manufacturer’s instructions. All samples were harvested at approximately 60 hours post-transfection. For lentiviral particle production, RRP6- and MTR4-expressing lentiviruses were produced in HEK293T cells by transfecting plasmids using calcium-phosphate and HeLa cells were transduced as described previously [7]. Lentiviruses expressing shRNAs (Sigma) were produced in 293T cells according to manufacturer’s instructions.

For HIV-1 infection, peripheral blood mononuclear cells (PBMCs) were isolated from buffy coats of healthy HIV negative donors using Ficoll density gradient (Eurobio) and CD4+ T-cells were isolated by negative selection (Stem Cell Research). Cells were then activated with anti-CD3/CD28 beads (Miltenyi) and IL2 (30 U/ml) for 3 days, then infected with HIV-1_BaL_ as previously described [21]. Three days post-infection, cells were transferred to media containing 10 U/ml IL2 and transduced with lentiviral particles expressing shRNAs. Viral release in the supernatant was quantified 7 days later by p24 ELISA (InGen).

To establish latently infected primary cells, PBMCs were isolated from buffy coats (Day 0) of healthy HIV negative donors using Ficoll density gradient and CD4+ T-cells were isolated by negative selection. Cells were activated by anti-CD3/CD28 + IL2 (30 U/ml) at day 1 as previously described [29] and infected with HIV-1_DuoFluo_ at day 4. Transduction with lentiviruses expressing shRNA constructs was performed at day 7 on previously sorted latently infected cells. Cells were maintained in cell culture medium containing 10 U/ml IL2. FACS analyses were performed at day 19. HIV-1_DuoFluo_ was obtained through the NIH AIDS Reagent Program, Division of AIDS, NIAID, NIH: Cat# 12595 DuoFluo (R7GEmC) from Drs. Vincenzo Calvanez and Eric Verdin [29].

### Small interfering RNAs and q-PCR oligonucleotides

Target sequences of double stranded RNA oligonucleotides used for RNAi (Eurofins MWG Operon or IDT) are shown in S3 Table. Sequences of PCR primers used in this study are shown in S4 Table.

### Protein complex purification

RRP6 and MTR4 complexes were purified from Dignam high salt nuclear extracts (https://dx.doi.org/10.17504/protocols.io.kh2ct8e) from HeLa-S3 cells stably expressing Flag-HA-RRP6 or Flag-HA-MTR4 by two-step affinity chromatography (10.17504/protocols.io.kgrctv6). Sequential Flag and HA immunoprecipitations were performed on equal amounts of proteins. Silver-staining was performed according to the manufacturer’s instruction (Silverquest, Invitrogen). Mass spectrometry was performed at Taplin facility, Harvard University, Boston, MA.

### Glycerol gradient analysis

One ml layers of glycerol (35% to 15%) were loaded in Ultraclear tubes (Beckman). RRP6-associated proteins were purified by affinity chromatography using Flag-beads (Sigma). Purified complexes were eluted by competition using Flag-peptides (Sigma). Flag-peptide-eluted material was resolved by size-exclusion chromatography. One mL of each glycerol buffer (final concentration 15 to 35%–20 mM Tris pH 7.5, 0.15 M KCl, 2.5 mM MgCl2, 0.05% NP-40, 0.1% Tween) was layered into centrifugation tubes (13 x 51 mm Ultra-Clear Tubes, Beckman). A linear gradient was obtained after 12 h of diffusion at 4°C. Flag elution from HeLa-Flag-HA-MTR4 immunoprecipitate was loaded on top of the glycerol gradient. Complexes were fractionated by ultracentrifugation in an SW 55Ti rotor (Beckman) at 40,000 rpm for 8 h at 4°C. 25 fractions of 200 μL were collected from top of the gradient. An equal volume of fractions was resolved by SDS-PAGE and immunoblotted with indicated antibodies.

### Immunoblot, luciferase assay and p24 ELISA

For immunoblot, protein extracts were obtained using RIPA buffer (50 mM Tris-HCl pH = 7.5, 150 mM NaCl, 1% NP40, 0.5% Sodium Deoxycholate, 0.1% SDS) supplemented with Complete protease inhibitor (Roche). Protein extracts were immunoblotted using the indicated primary antibodies (S2 Table) and anti-mouse or anti-rabbit IgG-linked HRP secondary antibodies (GE Healthcare) followed by ECL (ThermoFisher). Band intensities were quantified using ImageJ. Luciferase assay was performed according to the manufacturer’s instructions (Promega). P24 was quantified in cell culture supernatants using Innotest HIV Ag assay (InGen) according to manufacturer’s instructions.

### Quantitative RT-PCR

Total RNA was extracted from cells using TRIzol (ThermoFisher Scientific) according to the manufacturer’s instructions. Extracts were treated with DNase I (Promega) and reverse transcribed using SuperScript III First-Strand Synthesis System (ThermoFisher Scientific). RT products were amplified by real time PCR (Lightcycler, Roche) using Quanti Tect SYBR Green (Qiagen) with the indicated oligonucleotides. Q-PCR cycling conditions are available on request. Sequences of qPCR primers used in this study are shown in S4 Table.

### Nuclear run-on transcription assay

Run-on transcription was performed as described in https://dx.doi.org/10.17504/protocols.io.khxct7n. Run-on transcripts were reverse transcribed and quantified by PCR using the oligonucleotide pairs indicated. Results were normalized to the amount of GAPDH run-on transcript in the same sample.

### Chromatin immunoprecipitation (ChIP) and RNA-ChIP

HeLa LTR-luc cells were transfected as indicated in the figures. Following 64 hr incubation, cells were washed and harvested for cross-link ChIP, which was performed as described in https://dx.doi.org/10.17504/protocols.io.knkcvcw, or RNA-ChIP, which was performed as described previously [28] using the antibodies indicated. Samples were amplified by qPCR or RT-qPCR using the primer pairs indicated. An aliquot of chromatin was amplified in parallel and values obtained for immunoprecipitates were normalized to values for chromatin (% input).

For non-treated J-Lat cells, cells were harvested for cross-link ChIP using iDeal ChIP q-PCR kit (Diagenode) according to the manufacturer’s instructions. For J-lat cells that had been transduced with shRNAs as indicated in the figures, cells were harvested 7 days post-transduction and processed using LowCell ChIP kit (Diagneode) according to the manufacturer’s instructions.

### Flow cytometry analysis and sorting

J-Lat cells clone 10.6 (obtained from E. Verdin) were fixed in PBS containing 1% paraformaldehyde for 10 mins, washed with PBS and resuspended in fresh PBS. GFP fluorescence was measured with a MAQS Quant machine (Miltenyi Biotech). Electronic compensation was applied during analysis. Analysis was gated on live cells according to forward and side scatter. A gate (GFP positive) containing GFP-positive cells was drawn compared to untreated cells. For FACS analysis of HIV-1_DuoFluo_ infected PBMCs, control cells infected with pHRET (GFP-positive) or NL4-3 mCherry viral vector (Addgene#44965) were used to apply fluorescence compensation to the data. Data were collected on a Novocyte cytometer (Ozyme) and data were analyzed using FlowJo software (TreeStar).

## Supporting information

S1 Table List of interactants of Flag-HA-RRP6 or Flag-HA-MTR4 identified by mass spectrometry. Names of proteins shown in highlight are those identified by Lubas et al, 2011.
(PDF)

S2 Table List of antibodies used in this study. (TIFF)

S3 Table Sequences of siRNAs used in this study. (TIFF)

S4 Table Sequences of shRNAs used in this study. (TIFF)

S5 Table Sequences of qPCR primers used in this study. (TIFF)

S1 Fig Comparison of RRP6 and MTR4 interactomes. (A) Venn diagram showing the number of RRP6-interacting and MTR4-interacting proteins identified by the present study. (B) Venn diagram showing the number of RRP6-interacting proteins identified by Lubas et al (2011) compared to the present study. (C) Venn diagram showing the number of MTR4-interacting proteins identified by Lubas et al (2011) compared to the present study.
(TIFF)

S2 Fig ZFC3H1 and ZCCHC8 partially Co-Localize with RNAPII. Immunofluorescence microscopy analysis of HeLa LTR-luc cells stained with antibodies to ZFC3H1, ZCCHC8 and RNAPII and DAPI to visualize cell nuclei, as indicated. Scale bar represents 1 υm.
(TIFF)

S3 Fig Ablation of C1D, but not core exosome or DIS3, stimulates LTR activity. (A) HeLa-LTR-luc cells were transfected with siRNAs directed against C1D, MTR4 or a control siRNA (Scr) at the concentrations indicated. Cell extracts were harvested at 60 hr and analyzed by luciferase assay and immunoblotting using the antibodies indicated. Fold activation was calculated relative to the control transfection (Scr), which was attributed a value of 1. Graphs represent mean ± SEM obtained from 3 independent experiments (**P < 0.01, *P < 0.05, independent Student’s *t* test). (B) HeLa-LTR-luc cells were transfected with siRNAs directed against RRP6, EXOSC2/7/9, DIS3 or a control siRNA (Scr). Cell extracts were harvested at 60 hr and analyzed by luciferase assay and immunoblotting using the antibodies indicated. Fold activation was calculated relative to the control transfection (Scr), which was attributed a value of 1. Graphs represent mean ± SEM obtained from 3 independent experiments (**P < 0.01, NS indicates not significant, independent Student’s *t* test).
(TIFF)

S4 Fig MTR4, RRP6, ZFC3H1 or ZCCHC8 do not modulate expression of HIV-1 transcriptional Co-Activators. HeLa-LTR-luc cells were transfected with siRNAs directed against MTR4, RRP6, ZFC3H1 + ZCCHC8 or a control siRNA (Scr). Cell extracts were harvested at 60 hr post-transfection and analyzed by immunoblotting using the antibodies indicated.
(TIFF)

S5 Fig Detection of autofluorescence of shRNA-treated and α-CD3-CD28 stimulated PBMCs. (A) Activated PBMCs from healthy donors that were not infected with HIV-1_DuoFluo_ were transduced with lentiviral particles expressing the indicated shRNAs or stimulated using **α**-CD3-CD28, similar to Fig 8. Cells were analyzed by flow cytometry 7 days later. (B) An aliquot of cells shown in A was analyzed by immunoblotting using the indicated antibodies.
(TIFF)
