# Supplementary material for: Nuclear RNA surveillance complexes silence HIV-1 transcription
Source: PLoS Pathog. 2018 Mar 19;14(3):e1006950. doi: 10.1371/journal.ppat.1006950 (PMC5875879; doi:10.1371/journal.ppat.1006950)
Supplement: S1 Table — Names of proteins shown in highlight are those identified by Lubas et al, 2011. (PDF) [file ppat.1006950.s001.pdf]

S1 Table. List of interactants of Flag-HA RRP6 or Flag-HA-MTR4 identified by mass spectrometry. Names of proteins shown in highlight are those also identified by Lubas et al. 2011

	RRP6			MTR4	
**reference**	**Gene Symbol**	**UniProtID**	**reference**	**Gene Symbol**	**UniProtID**
ELYS_HUMAN	AHCTF1	Q8WYP5	AKAP8_HUMAN	AKAP8	O43823
AKAP8_HUMAN	AKAP8	O43823	AKP8L_HUMAN	AKAP8L	Q9ULX6
AKP8L_HUMAN	AKAP8L	Q9ULX6	AL3A1_HUMAN	ALDH3A1	P30838
ALDOA_HUMAN	ALDOA	P04075	AL3A2_HUMAN	ALDH3A2	P51648
ANXA2_HUMAN	ANXA2	P07355	ALDOA_HUMAN	ALDOA	P04075
BAG2_HUMAN	BAG2	O95816	ALDOC_HUMAN	ALDOC	P09972
C1D_HUMAN	C1D	Q13901	ANXA2_HUMAN	ANXA2	P07355
PYR1_HUMAN	CAD	P27708	AXA2L_HUMAN	ANXA2P2	A6NMY6
CART_HUMAN	CARTPT	Q16568	DX39B_HUMAN	BAT1	Q13838
TCPB_HUMAN	CCT2	P78371	C1D_HUMAN	C1D	Q13901
TCPG_HUMAN	CCT3	P49368	CALL3_HUMAN	CALML3	P27482
TCPD_HUMAN	CCT4	P50991	CALL5_HUMAN	CALML5	Q9NZT1
TCPH_HUMAN	CCT7	Q99832	CALX_HUMAN	CANX	P27824
TCPQ_HUMAN	CCT8	P50990	CASPE_HUMAN	CASP14	P31944
CDK9_HUMAN	CDK9	P50750	CCAR2_HUMAN	CCAR2	Q8N163
CE170_HUMAN	CEP170	Q5SW79	CDC73_HUMAN	CDC73	Q6P1J9
CAF1B_HUMAN	CHAF1B	Q13112	CATD_HUMAN	CTSD	P07339
CHD9_HUMAN	CHD9	Q3L8U1	DAXX_HUMAN	DAXX	Q9UER7
DDX17_HUMAN	DDX17	Q92841	DDX17_HUMAN	DDX17	Q92841
DD19A_HUMAN	DDX19A	Q9NUU7	DD19B_HUMAN	DDX19B	Q9UMR2
DDX21_HUMAN	DDX21	Q9NR30	DDX21_HUMAN	DDX21	Q9NR30
DDX31_HUMAN	DDX31	Q9H8H2	DDX3X_HUMAN	DDX3X	O00571
DX39A_HUMAN	DDX39A	O00148	DDX46_HUMAN	DDX46	Q7L014
DDX3X_HUMAN	DDX3X	O00571	DDX47_HUMAN	DDX47	Q9H0S4
DDX41_HUMAN	DDX41	Q9UJV9	DDX5_HUMAN	DDX5	P17844
DDX5_HUMAN	DDX5	P17844	DHX15_HUMAN	DHX15	O43143
DDX52_HUMAN	DDX52	Q9Y2R4	DHX9_HUMAN	DHX9	Q08211
DHX15_HUMAN	DHX15	O43143	DYN2_HUMAN	DNM2	P50570
Q7Z3W9_HUMAN	DKFZp686G18	Q7Z3W9	EF1A1_HUMAN	EEF1A1	P68104
DNJA1_HUMAN	DNAJA1	P31689	EF1A2_HUMAN	EEF1A2	Q05639
TDIF2_HUMAN	DNTTIP2	Q5QJE6	EF2_HUMAN	EEF2	P13639
DC1L1_HUMAN	DYNC1LI1	Q9Y6G9	IF4A1_HUMAN	EIF4A1	P60842
EBP2_HUMAN	EBNA1BP2	Q99848	IF4A3_HUMAN	EIF4A3	P38919
EF1A3_HUMAN	EEF1A1P5	Q5VTE0	IF6_HUMAN	EIF6	P56537
EF1A2_HUMAN	EEF1A2	Q05639	ELAV1_HUMAN	ELAVL1	Q15717
IF4A3_HUMAN	EIF4A3	P38919	ENOA_HUMAN	ENO1	P06733
IF6_HUMAN	EIF6	P56537	ENOG_HUMAN	ENO2	P09104
ELAV1_HUMAN	ELAVL1	Q15717	EX3L1_HUMAN	EXOC3L1	Q86VI1
EWS_HUMAN	EWSR1	Q01844	EXOS1_HUMAN	EXOSC1	Q9Y3B2
EXOS1_HUMAN	EXOSC1	Q9Y3B2	EXOSX_HUMAN	EXOSC10	Q01780
EXOSX_HUMAN	EXOSC10	Q01780	EXOS2_HUMAN	EXOSC2	Q13868
EXOS2_HUMAN	EXOSC2	Q13868	EXOS3_HUMAN	EXOSC3	Q9NQT5
EXOS3_HUMAN	EXOSC3	Q9NQT5	EXOS4_HUMAN	EXOSC4	Q9NPD3
EXOS4_HUMAN	EXOSC4	Q9NPD3	EXOS5_HUMAN	EXOSC5	Q9NQT4
EXOS5_HUMAN	EXOSC5	Q9NQT4	EXOS6_HUMAN	EXOSC6	Q5RKV6
EXOS6_HUMAN	EXOSC6	Q5RKV6	EXOS7_HUMAN	EXOSC7	Q15024
EXOS7_HUMAN	EXOSC7	Q15024	EXOS8_HUMAN	EXOSC8	Q96B26
EXOS8_HUMAN	EXOSC8	Q96B26	EXOS9_HUMAN	EXOSC9	Q06265
EXOS9_HUMAN	EXOSC9	Q06265	FA98A_HUMAN	FAM98A	Q8NCA5
FAS_HUMAN	FASN	P49327	FUS_HUMAN	FUS	P35637
FIP1_HUMAN	FIP1L1	Q6UN15	FXR1_HUMAN	FXR1	P51114

FWCH1_HUMAN	FLYWCH1	Q4VC44
FUBP3_HUMAN	FUBP3	Q96I24
FUS_HUMAN	FUS	P35637
GCN1_HUMAN	GCN1	Q92616
GNL3L_HUMAN	GNL3L	Q9NVN8
TF3C1_HUMAN	GTF3C1	Q12789
TF3C4_HUMAN	GTF3C4	Q9UKN8
TF3C5_HUMAN	GTF3C5	Q9Y5Q8
NOG1_HUMAN	GTPBP4	Q9BZE4
HDAC2_HUMAN	HDAC2	Q92769
HEAT1_HUMAN	HEATR1	Q9H583
H11_HUMAN	HIST1H1A	Q02539
H12_HUMAN	HIST1H1C	P16403
H2A1A_HUMAN	HIST1H2AA	Q96QV6
H4_HUMAN	HIST1H4A	P62805
ROA2_HUMAN	HNRNPA2B1	P22626
HNRC1_HUMAN	HNRNPCL1	O60812
HNRPD_HUMAN	HNRNPD	Q14103
HNRPF_HUMAN	HNRNPF	P52597
HNRH1_HUMAN	HNRNPH1	P31943
HNRPK_HUMAN	HNRNPK	P61978
HNRPL_HUMAN	HNRNPL	P14866
HNRPM_HUMAN	HNRNPM	P52272
HPT_HUMAN	HP	P00738
HS90A_HUMAN	HSP90AA1	P07900
HS90B_HUMAN	HSP90AB1	P08238
ENPL_HUMAN	HSP90B1	P14625
HS71A_HUMAN	HSPA1A	P0DMV8
HS71L_HUMAN	HSPA1L	P34931
HSP72_HUMAN	HSPA2	P54652
GRP78_HUMAN	HSPA5	P11021
HSP76_HUMAN	HSPA6	P17066
HSP7C_HUMAN	HSPA8	P11142
GRP75_HUMAN	HSPA9	P38646
HSPB1_HUMAN	HSPB1	P04792
CH60_HUMAN	HSPD1	P10809
ILF2_HUMAN	ILF2	Q12905
IRX4_HUMAN	IRX4	P78413
PLAK_HUMAN	JUP	P14923
KHDR1_HUMAN	KHDRBS1	Q07666
LAS1L_HUMAN	LAS1L	Q9Y4W2
LBHD1_HUMAN	LBHD1	Q9BQE6
LMNA_HUMAN	LMNA	P02545
LMNB1_HUMAN	LMNB1	P20700
LRC40_HUMAN	LRRC40	Q9H9A6
LSG1_HUMAN	LSG1	Q9H089
MAGD1_HUMAN	MAGED1	Q9Y5V3
MAGD2_HUMAN	MAGED2	Q9UNF1
MAP7_HUMAN	MAP7	Q14244
JIP3_HUMAN	MAPK8IP3	Q9UPT6
MATR3_HUMAN	MATR3	P43243
MCM7_HUMAN	MCM7	P33993
MGAP_HUMAN	MGA	Q8IWI9
MISP_HUMAN	MISP	Q8IVT2
MPH6_HUMAN	MPHOSPH6	Q99547
MSH6_HUMAN	MSH6	P52701

G3P_HUMAN	GAPDH	P04406
GCN1_HUMAN	GCN1	Q92616
GBB2_HUMAN	GNB2	P62879
GSTP1_HUMAN	GSTP1	P09211
NOG1_HUMAN	GTPBP4	Q9BZE4
H2AV_HUMAN	H2AFV	Q71UI9
HEAT1_HUMAN	HEATR1	Q9H583
H12_HUMAN	HIST1H1C	P16403
H2A1B_HUMAN	HIST1H2AB	P04908
H4_HUMAN	HIST1H4A	P62805
H2B2E_HUMAN	HIST2H2BE	Q16778
HNRPC_HUMAN	HNRNPC	P07910
HNRC1_HUMAN	HNRNPCL1	O60812
HNRPD_HUMAN	HNRNPD	Q14103
HNRPF_HUMAN	HNRNPF	P52597
HNRH1_HUMAN	HNRNPH1	P31943
HNRH3_HUMAN	HNRNPH3	P31942
HNRPK_HUMAN	HNRNPK	P61978
HNRPL_HUMAN	HNRNPL	P14866
HNRPM_HUMAN	HNRNPM	P52272
HNRPU_HUMAN	HNRNPU	Q00839
HNRL1_HUMAN	HNRNPUL1	Q9BUJ2
HNRL2_HUMAN	HNRNPUL2	Q1KMD3
HS90A_HUMAN	HSP90AA1	P07900
HS71A_HUMAN	HSPA1A	P0DMV8
GRP78_HUMAN	HSPA5	P11021
HSP77_HUMAN	HSPA7	P48741
HSP7C_HUMAN	HSPA8	P11142
HSPB1_HUMAN	HSPB1	P04792
IDE_HUMAN	IDE	P14735
ILF2_HUMAN	ILF2	Q12905
ILK_HUMAN	ILK	Q13418
ILVBL_HUMAN	ILVBL	A1L0T0
IQGA1_HUMAN	IQGAP1	P46940
KHDR1_HUMAN	KHDRBS1	Q07666
LDHA_HUMAN	LDHA	P00338
LDHB_HUMAN	LDHB	P07195
LEG7_HUMAN	LGALS7	P47929
LMNA_HUMAN	LMNA	P02545
LMO7_HUMAN	LMO7	Q8WWI1
LYAR_HUMAN	LYAR	Q9NX58
MATR3_HUMAN	MATR3	P43243
MPH6_HUMAN	MPHOSPH6	Q99547
MSH2_HUMAN	MSH2	P43246
MSH6_HUMAN	MSH6	P52701
MYCBP_HUMAN	MYCBP	Q99417
NCBP1_HUMAN	NCBP1	Q09161
NCBP2_HUMAN	NCBP2	P52298
NPM_HUMAN	NPM1	P06748
NRDE2_HUMAN	NRDE2	Q9H7Z3
NUMA1_HUMAN	NUMA1	Q14980
PABP2_HUMAN	PABPN1	Q86U42
PCBP1_HUMAN	PCBP1	Q15365
PCBP2_HUMAN	PCBP2	Q15366
PDS5A_HUMAN	PDS5A	Q29RF7
PEBP1_HUMAN	PEBP1	P30086

MYCBP_HUMAN	MYCBP	Q99417
NACC1_HUMAN	NACC1	Q96RE7
NACC2_HUMAN	NACC2	Q96BF6
NP1L1_HUMAN	NAP1L1	P55209
NCBP1_HUMAN	NCBP1	Q09161
NUCL_HUMAN	NCL	P19338
NOL11_HUMAN	NOL11	Q9H8H0
NOL9_HUMAN	NOL9	Q5SY16
NOP56_HUMAN	NOP56	O00567
NOP58_HUMAN	NOP58	Q9Y2X3
NPM_HUMAN	NPM1	P06748
GCR_HUMAN	NR3C1	P04150
NUMA1_HUMAN	NUMA1	Q14980
NVL_HUMAN	NVL	O15381
PABP2_HUMAN	PABPN1	Q86U42
PCBP2_HUMAN	PCBP2	Q15366
PFKAL_HUMAN	PFKL	P17858
PFKAP_HUMAN	PFKP	Q01813
PPIA_HUMAN	PPIA	P62937
PRDX6_HUMAN	PRDX6	P30041
PRKDC_HUMAN	PRKDC	P78527
ANM5_HUMAN	PRMT5	O14744
PRP8_HUMAN	PRPF8	Q6P2Q9
PRS6B_HUMAN	PSMC4	P43686
PSMD5_HUMAN	PSMD5	Q16401
PTBP1_HUMAN	PTBP1	P26599
PWP2_HUMAN	PWP2	Q15269
QSER1_HUMAN	QSER1	Q2KHR3
RASL1_HUMAN	RASAL1	O95294
RBM14_HUMAN	RBM14	Q96PK6
RBM39_HUMAN	RBM39	Q14498
RBM7_HUMAN	RBM7	Q9Y580
RFC2_HUMAN	RFC2	P35250
RFC3_HUMAN	RFC3	P40938
RFC4_HUMAN	RFC4	P35249
RIF1_HUMAN	RIF1	Q5UIP0
RNF12_HUMAN	RLIM	Q9NVW2
RN219_HUMAN	RNF219	Q5W0B1
RL11_HUMAN	RPL11	P62913
RL12_HUMAN	RPL12	P30050
RL13_HUMAN	RPL13	P26373
RL13A_HUMAN	RPL13A	P40429
RL14_HUMAN	RPL14	P50914
RL18_HUMAN	RPL18	Q07020
RL19_HUMAN	RPL19	P84098
RL22_HUMAN	RPL22	P35268
RL23_HUMAN	RPL23	P62829
RL24_HUMAN	RPL24	P83731
RL27A_HUMAN	RPL27A	P46776
RL3_HUMAN	RPL3	P39023
RL30_HUMAN	RPL30	P62888
RL31_HUMAN	RPL31	P62899
RL34_HUMAN	RPL34	P49207
RL35_HUMAN	RPL35	P42766
RL38_HUMAN	RPL38	P63173
RL4_HUMAN	RPL4	P36578

PELP1_HUMAN	PELP1	Q8IZL8
PFKAP_HUMAN	PFKP	Q01813
PGAM2_HUMAN	PGAM2	P15259
PKP1_HUMAN	PKP1	Q13835
PPIA_HUMAN	PPIA	P62937
PTBP1_HUMAN	PTBP1	P26599
QSER1_HUMAN	QSER1	Q2KHR3
RB11B_HUMAN	RAB11B	Q15907
RAB1A_HUMAN	RAB1A	P62820
RAB7A_HUMAN	RAB7A	P51149
RACK1_HUMAN	RACK1	P63244
RBM10_HUMAN	RBM10	P98175
RBM14_HUMAN	RBM14	Q96PK6
RBM39_HUMAN	RBM39	Q14498
RBM4_HUMAN	RBM4	Q9BWF3
RBM7_HUMAN	RBM7	Q9Y580
RDH11_HUMAN	RDH11	Q8TC12
RFC2_HUMAN	RFC2	P35250
RFC5_HUMAN	RFC5	P40937
RIF1_HUMAN	RIF1	Q5UIP0
RL11_HUMAN	RPL11	P62913
RL12_HUMAN	RPL12	P30050
RL18_HUMAN	RPL18	Q07020
RL19_HUMAN	RPL19	P84098
RL22_HUMAN	RPL22	P35268
RL24_HUMAN	RPL24	P83731
RL27A_HUMAN	RPL27A	P46776
RL3_HUMAN	RPL3	P39023
RL31_HUMAN	RPL31	P62899
RL38_HUMAN	RPL38	P63173
RL4_HUMAN	RPL4	P36578
RL5_HUMAN	RPL5	P46777
RL7_HUMAN	RPL7	P18124
RL7A_HUMAN	RPL7A	P62424
RL8_HUMAN	RPL8	P62917
RL9_HUMAN	RPL9	P32969
RLA0_HUMAN	RPLP0	P05388
RLA1_HUMAN	RPLP1	P05386
RLA2_HUMAN	RPLP2	P05387
RPN1_HUMAN	RPN1	P04843
RS13_HUMAN	RPS13	P62277
RS14_HUMAN	RPS14	P62263
RS15A_HUMAN	RPS15A	P62244
RS16_HUMAN	RPS16	P62249
RS18_HUMAN	RPS18	P62269
RS19_HUMAN	RPS19	P39019
RS2_HUMAN	RPS2	P15880
RS20_HUMAN	RPS20	P60866
RS3_HUMAN	RPS3	P23396
RS3A_HUMAN	RPS3A	P61247
RS4X_HUMAN	RPS4X	P62701
RS6_HUMAN	RPS6	P62753
RS7_HUMAN	RPS7	P62081
RS8_HUMAN	RPS8	P62241
RUVB1_HUMAN	RUVBL1	Q9Y265
RUVB2_HUMAN	RUVBL2	Q9Y230

RL6_HUMAN	RPL6	Q02878
RL7_HUMAN	RPL7	P18124
RL8_HUMAN	RPL8	P62917
RL9_HUMAN	RPL9	P32969
RLA0_HUMAN	RPLP0	P05388
RLA1_HUMAN	RPLP1	P05386
RLA2_HUMAN	RPLP2	P05387
RS13_HUMAN	RPS13	P62277
RS14_HUMAN	RPS14	P62263
RS15A_HUMAN	RPS15A	P62244
RS16_HUMAN	RPS16	P62249
RS182_HUMAN	RPS18	P62269
RS19_HUMAN	RPS19	P39019
RS2_HUMAN	RPS2	P15880
RS21_HUMAN	RPS21	P63220
RS23_HUMAN	RPS23	P62266
RS24_HUMAN	RPS24	P62847
RS25_HUMAN	RPS25	P62851
RS26_HUMAN	RPS26	P62854
RS28_HUMAN	RPS28	P62857
RS30_HUMAN	RPS30	P62861
RS7_HUMAN	RPS7	P62081
RS8_HUMAN	RPS8	P62241
RS9_HUMAN	RPS9	P46781
RSSA_HUMAN	RPSA	P08865
RUVB1_HUMAN	RUVBL1	Q9Y265
RUVB2_HUMAN	RUVBL2	Q9Y230
SHC1_HUMAN	SHC1	P29353
SK2L2_HUMAN	SKIV2L2	P42285
U520_HUMAN	SNRNP200	O75643
SMD2_HUMAN	SNRPD2	P62316
SMD3_HUMAN	SNRPD3	P62318
SPA5L_HUMAN	SPATA5L1	Q9BVQ7
SPTB2_HUMAN	SPTBN1	Q01082
SYNEM_HUMAN	SYNM	O15061
RBP56_HUMAN	TAF15	Q92804
TBL3_HUMAN	TBL3	Q12788
HTF4_HUMAN	TCF12	Q99081
TCPA_HUMAN	TCP1	P17987
LAP2A_HUMAN	TMPO	P42166
TOP2A_HUMAN	TOP2A	P11388
TRI29_HUMAN	TRIM29	Q14134
PCH2_HUMAN	TRIP13	Q15645
SEN54_HUMAN	TSEN54	Q7Z6J9
THIO_HUMAN	TXN	P10599
UBR5_HUMAN	UBR5	O95071
UBP11_HUMAN	USP11	P51784
UTP18_HUMAN	UTP18	Q9Y5J1
UTP20_HUMAN	UTP20	O75691
VIME_HUMAN	VIM	P08670
WDR18_HUMAN	WDR18	Q9BV38
WDR3_HUMAN	WDR3	Q9UNX4
WDR36_HUMAN	WDR36	Q8NI36
WDR75_HUMAN	WDR75	Q8IWA0
MEP50_HUMAN	WDR77	Q9BQA1
WRIP1_HUMAN	WRNIP1	Q96S55

SPB13_HUMAN	SERPINB13	Q9UIV8
SPB3_HUMAN	SERPINB3	P29508
SPB4_HUMAN	SERPINB4	P48594
SPB5_HUMAN	SERPINB5	P36952
SF3B1_HUMAN	SF3B1	O75533
SK2L2_HUMAN	SKIV2L2	P42285
4F2_HUMAN	SLC3A2	P08195
LAT1_HUMAN	SLC7A5	Q01650
SMC4_HUMAN	SMC4	Q9NTJ3
U520_HUMAN	SNRNP200	O75643
SPTB2_HUMAN	SPTBN1	Q01082
SRRT_HUMAN	SRRT	Q9BXP5
SSRA_HUMAN	SSR1	P43307
SSRG_HUMAN	SSR3	Q9UNL2
STK38_HUMAN	STK38	Q15208
SUGP2_HUMAN	SUGP2	Q8IX01
SYMPK_HUMAN	SYMPK	Q92797
TAF12_HUMAN	TAF12	Q16514
TADBP_HUMAN	TARDBP	Q13148
TFR1_HUMAN	TFRC	P02786
A0A024RBE7_HU	TMPO	A0A024RBE7
TPIS_HUMAN	TPI1	P60174
TREX2_HUMAN	TREX2	Q9BQ50
THIO_HUMAN	TXN	P10599
UBR5_HUMAN	UBR5	O95071
URB2_HUMAN	URB2	Q14146
VIME_HUMAN	VIM	P08670
VSIG8_HUMAN	VSIG8	Q5VU13
XPO1_HUMAN	XPO1	O14980
XRN2_HUMAN	XRN2	Q9H0D6
YBOX1_HUMAN	YBX1	P67809
YBOX3_HUMAN	YBX3	P16989
1433E_HUMAN	YWHAE	P62258
1433G_HUMAN	YWHAG	P61981
ZC3HE_HUMAN	ZC3H14	Q6PJT7
ZCH18_HUMAN	ZC3H18	Q86VM9
ZCHC8_HUMAN	ZCCHC8	Q6NZY4
ZC3H1_HUMAN	ZFC3H1	O60293
ZN326_HUMAN	ZNF326	Q5BKZ1

XRCC5_HUMAN	XRCC5	P13010
XRCC6_HUMAN	XRCC6	P12956
1433B_HUMAN	YWHAB	P31946
1433E_HUMAN	YWHAE	P62258
1433G_HUMAN	YWHAG	P61981
ZBT20_HUMAN	ZBTB20	Q9HC78
ZBT24_HUMAN	ZBTB24	O43167
ZCH18_HUMAN	ZC3H18	Q86VM9
ZCHC8_HUMAN	ZCCHC8	Q6NZY4
ZC3H1_HUMAN	ZFC3H1	O60293
ZN106_HUMAN	ZNF106	Q9H2Y7
ZN281_HUMAN	ZNF281	Q9Y2X9
ZN326_HUMAN	ZNF326	Q5BKZ1
ZN644_HUMAN	ZNF644	Q9H582
Q59F66_HUMAN		Q59F66
Q9P1K8_HUMAN		Q9P1K8
