# Supplementary figures and images for: Nuclear RNA surveillance complexes silence HIV-1 transcription
Source: PLoS Pathog. 2018 Mar 19;14(3):e1006950. doi: 10.1371/journal.ppat.1006950 (PMC5875879; doi:10.1371/journal.ppat.1006950)

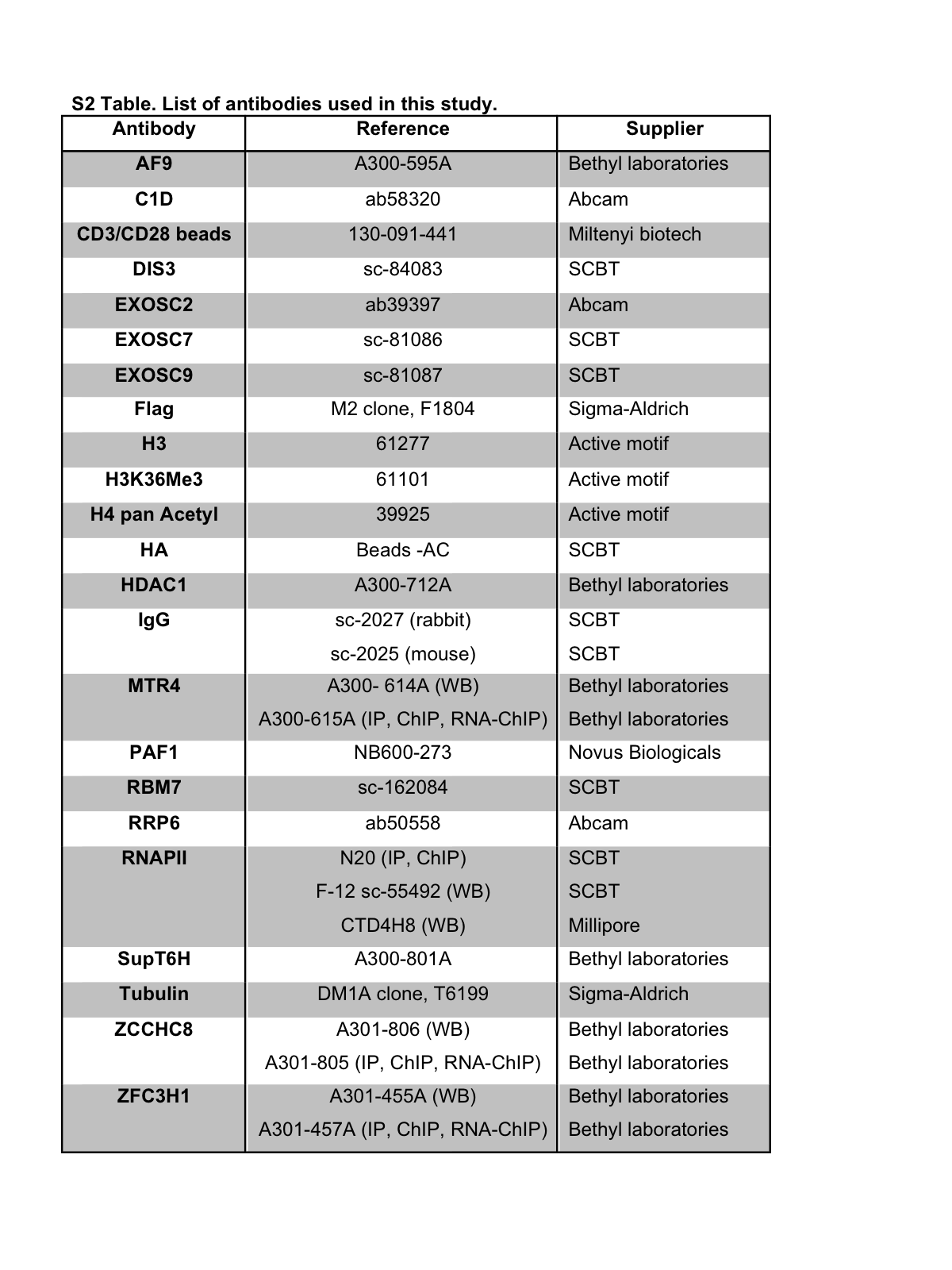

Supplement: S2 Table — (TIFF) [file ppat.1006950.s002.tiff]

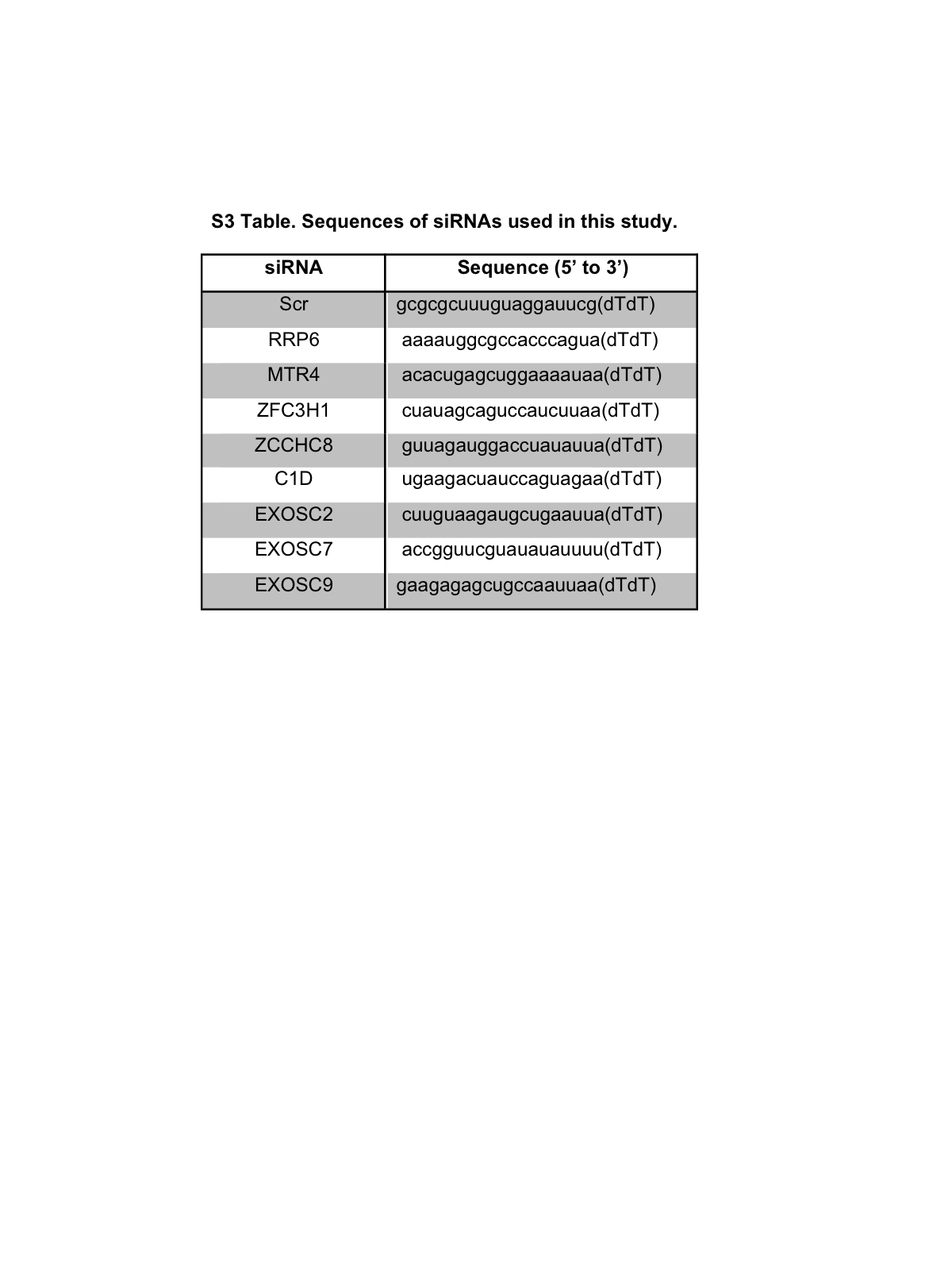

Supplement: S3 Table — (TIFF) [file ppat.1006950.s003.tiff]

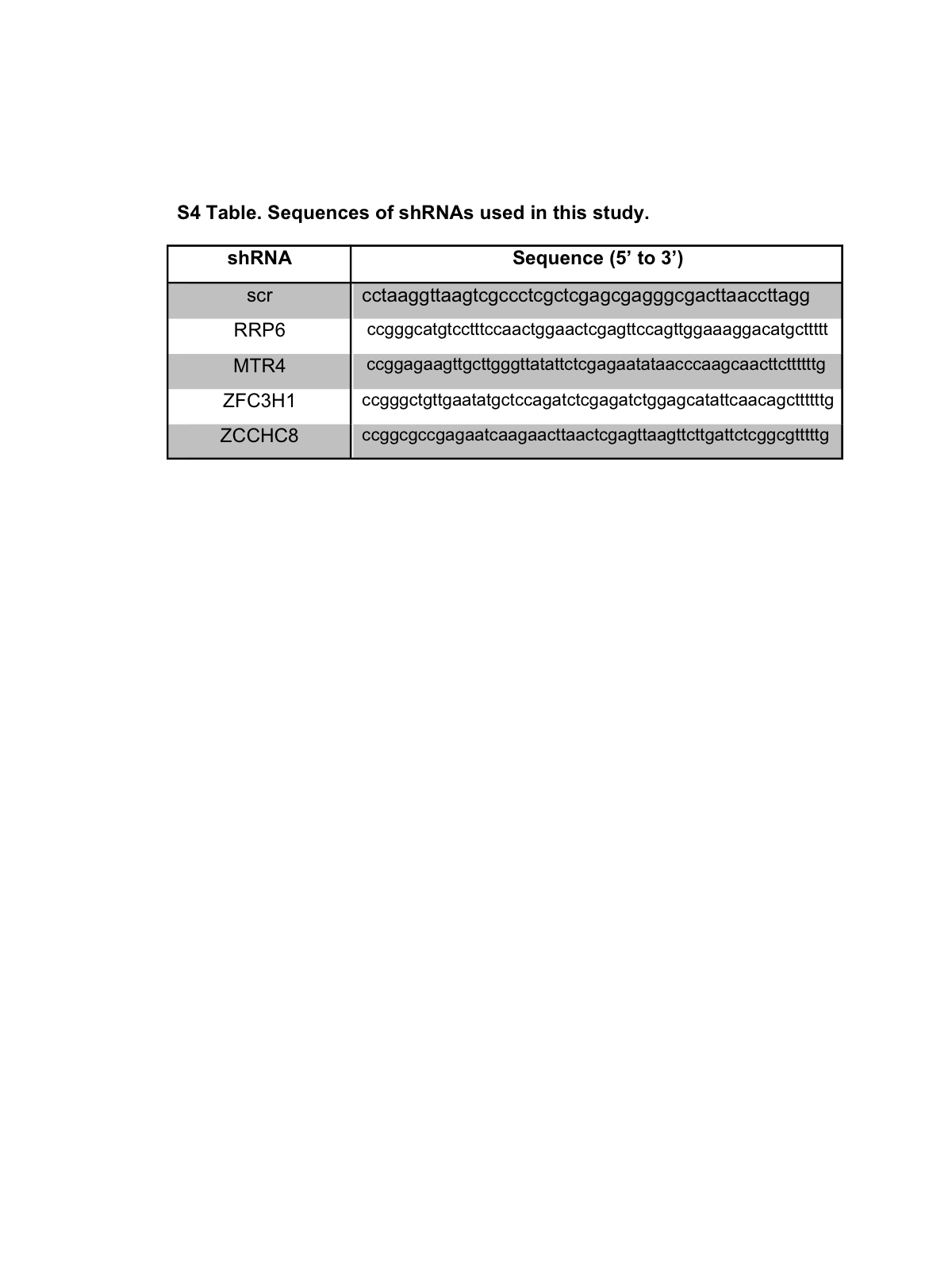

Supplement: S4 Table — (TIFF) [file ppat.1006950.s004.tiff]

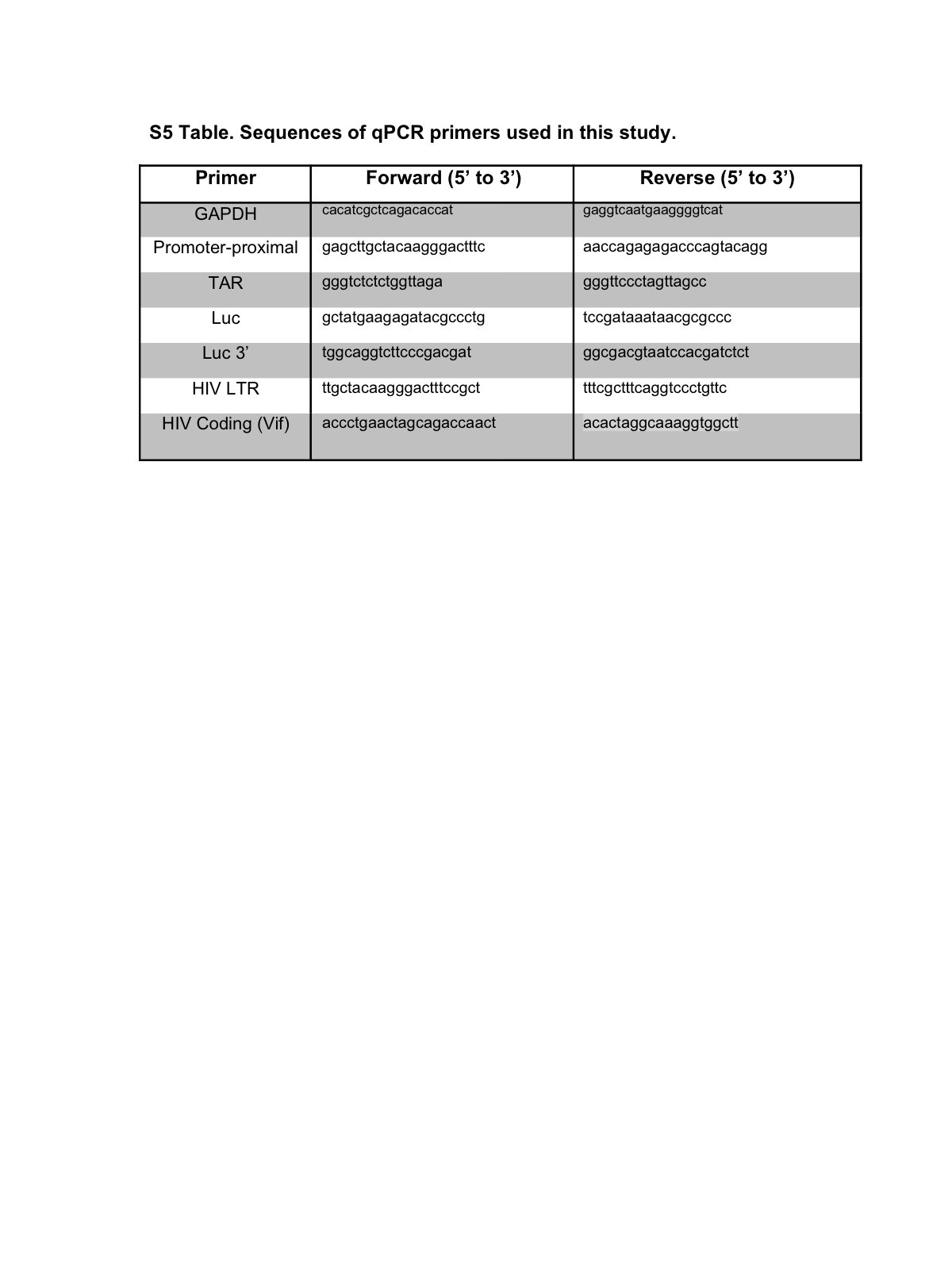

Supplement: S5 Table — (TIFF) [file ppat.1006950.s005.tiff]

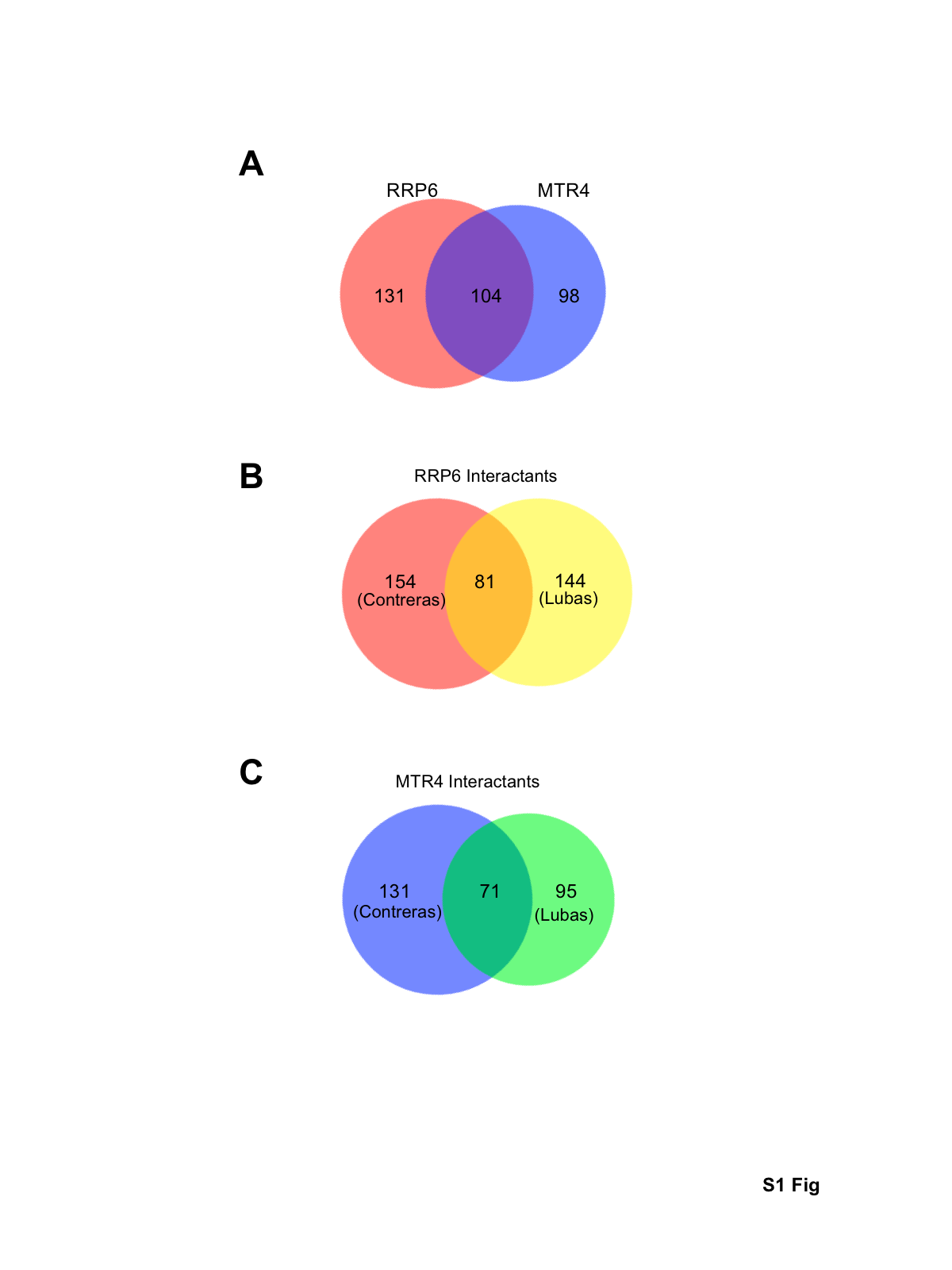

Supplement: S1 Fig — (A) Venn diagram showing the number of RRP6-interacting and MTR4-interacting proteins identified by the present study. (B) Venn diagram showing the number of RRP6-interacting proteins identified by Lubas et al (2011) compared to the present study. (C) Venn diagram showing the number of MTR4-interacting proteins identified by Lubas et al (2011) compared to the present study. (TIFF) [file ppat.1006950.s006.tiff]

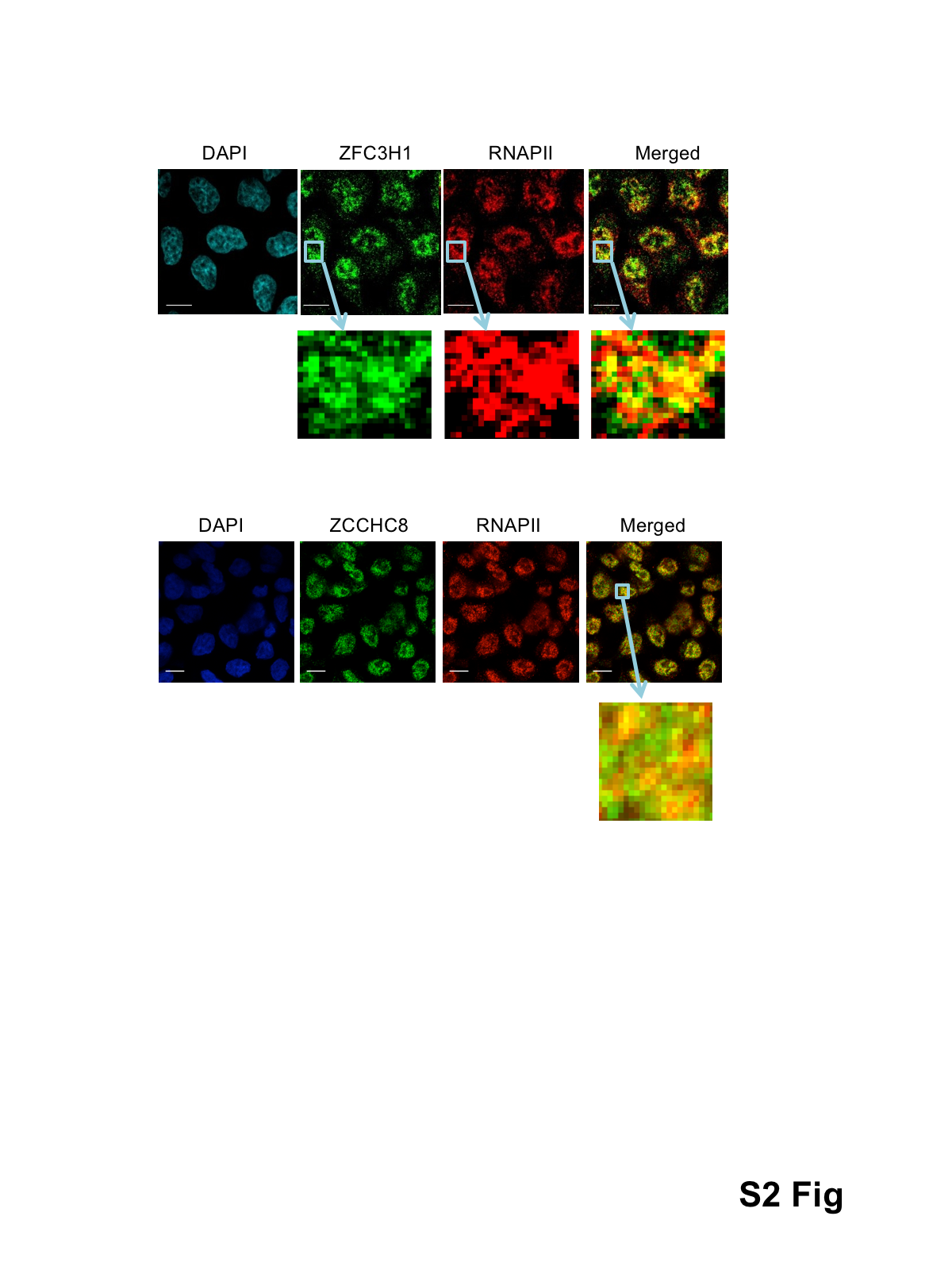

Supplement: S2 Fig — Immunofluorescence microscopy analysis of HeLa LTR-luc cells stained with antibodies to ZFC3H1, ZCCHC8 and RNAPII and DAPI to visualize cell nuclei, as indicated. Scale bar represents 1 υm. (TIFF) [file ppat.1006950.s007.tiff]

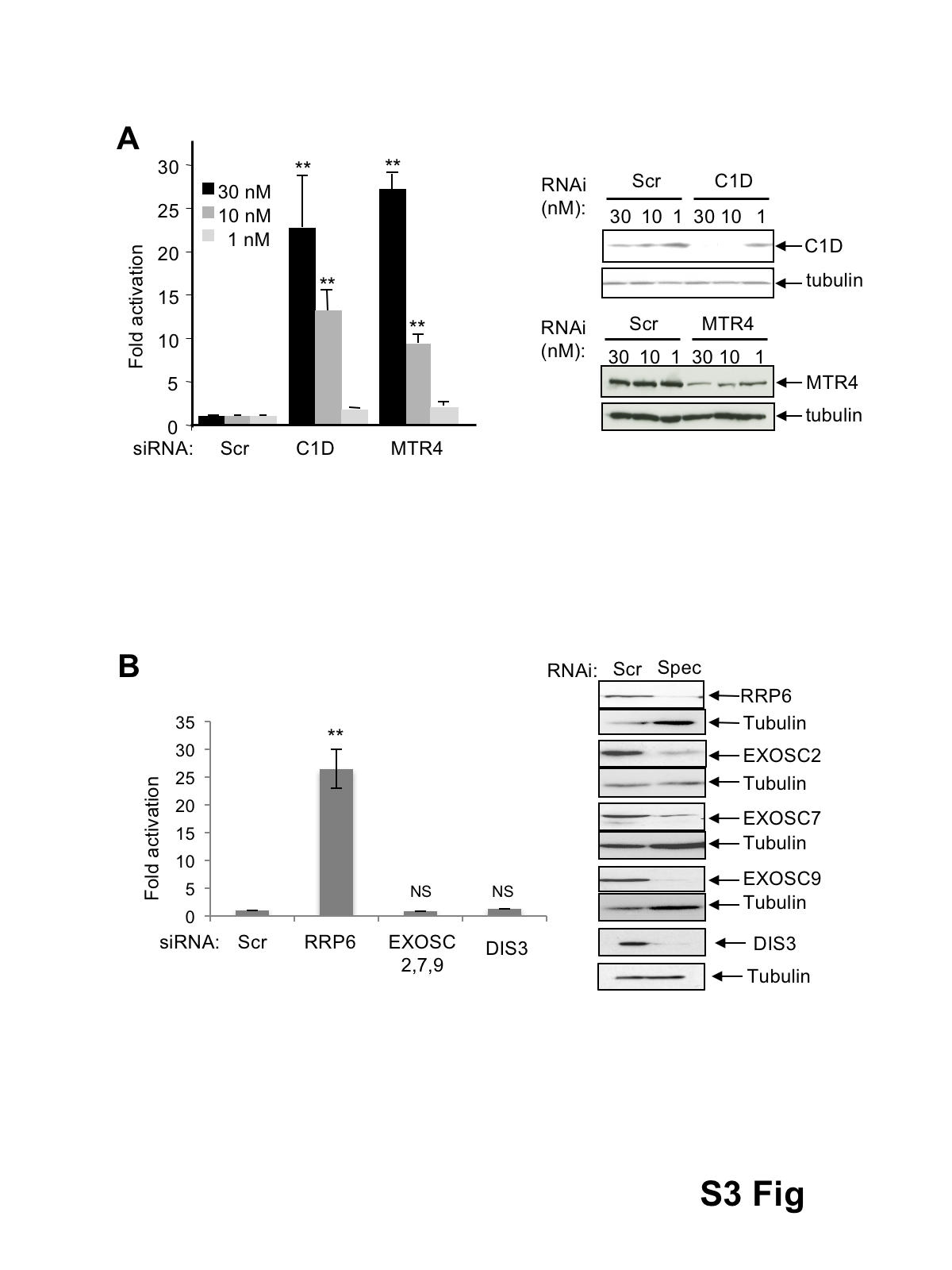

Supplement: S3 Fig — (A) HeLa-LTR-luc cells were transfected with siRNAs directed against C1D, MTR4 or a control siRNA (Scr) at the concentrations indicated. Cell extracts were harvested at 60 hr and analyzed by luciferase assay and immunoblotting using the antibodies indicated. Fold activation was calculated relative to the control transfection (Scr), which was attributed a value of 1. Graphs represent mean ± SEM obtained from 3 independent experiments (**P < 0.01, *P < 0.05, independent Student’s t test). (B) HeLa-LTR-luc cells were transfected with siRNAs directed against RRP6, EXOSC2/7/9, DIS3 or a control siRNA (Scr). Cell extracts were harvested at 60 hr and analyzed by luciferase assay and immunoblotting using the antibodies indicated. Fold activation was calculated relative to the control transfection (Scr), which was attributed a value of 1. Graphs represent mean ± SEM obtained from 3 independent experiments (**P < 0.01, NS indicates not significant, independent Student’s t test). (TIFF) [file ppat.1006950.s008.tiff]

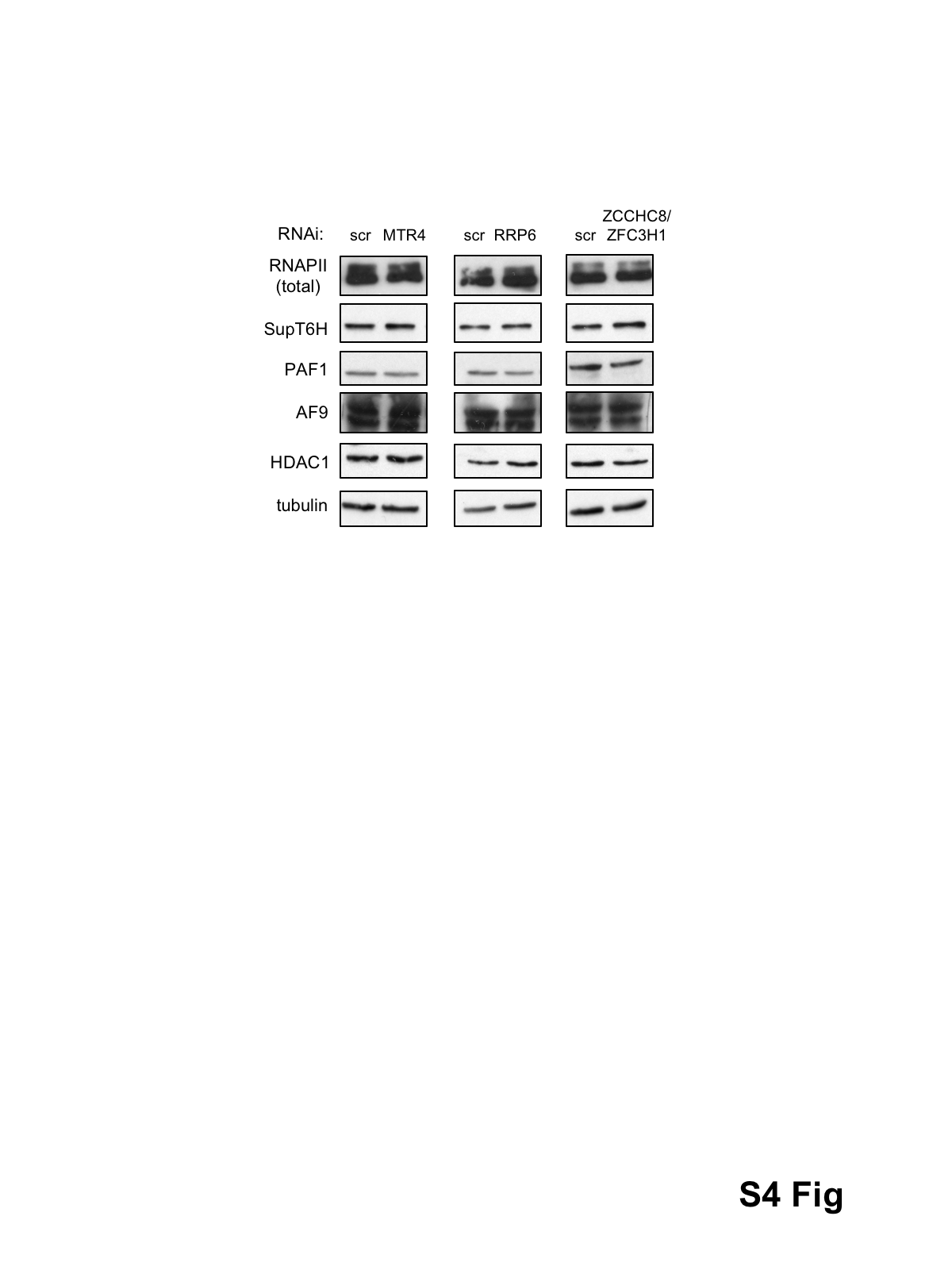

Supplement: S4 Fig — HeLa-LTR-luc cells were transfected with siRNAs directed against MTR4, RRP6, ZFC3H1 + ZCCHC8 or a control siRNA (Scr). Cell extracts were harvested at 60 hr post-transfection and analyzed by immunoblotting using the antibodies indicated. (TIFF) [file ppat.1006950.s009.tiff]

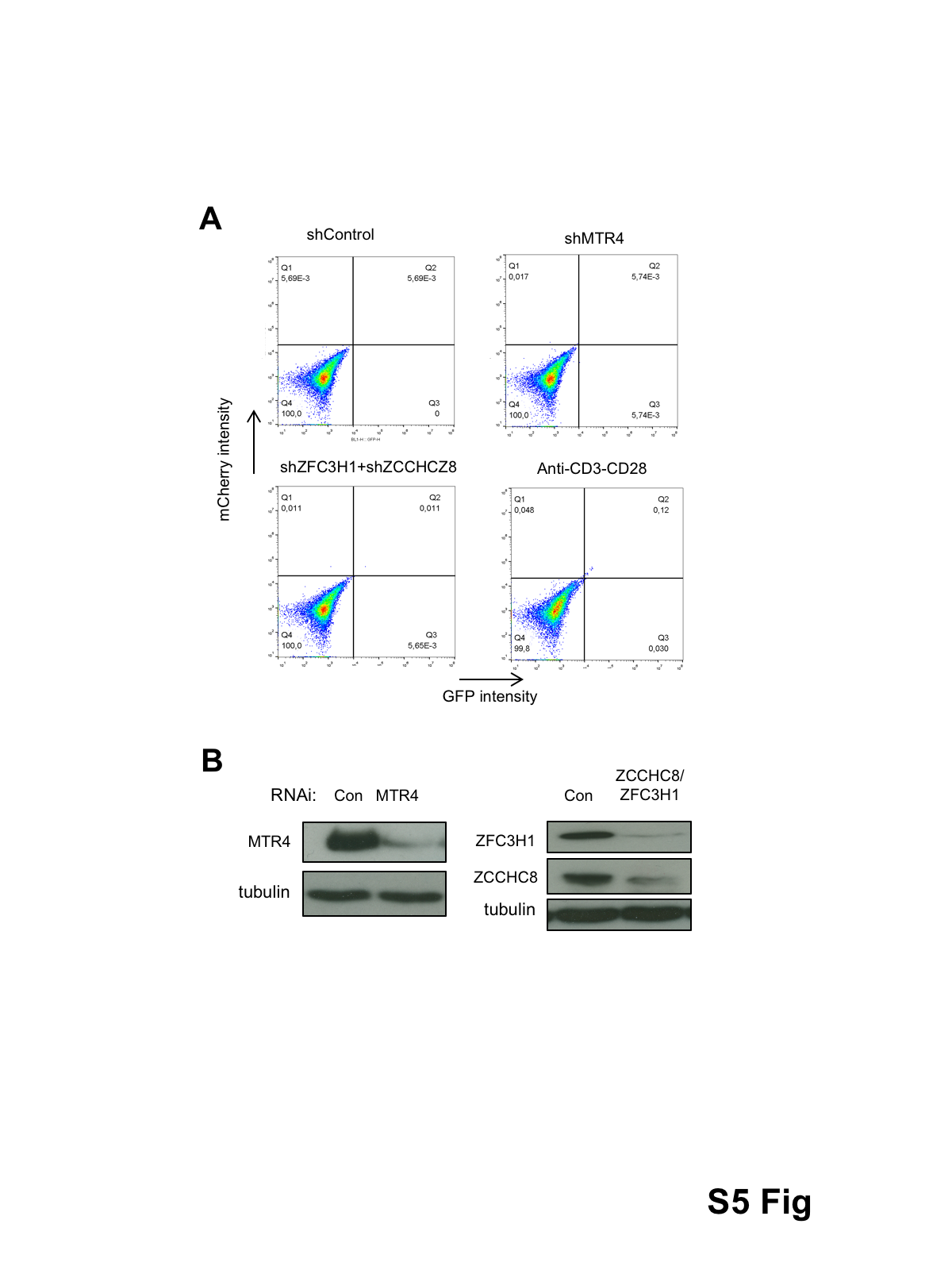

Supplement: S5 Fig — (A) Activated PBMCs from healthy donors that were not infected with HIV-1DuoFluo were transduced with lentiviral particles expressing the indicated shRNAs or stimulated using α-CD3-CD28, similar to Fig 8. Cells were analyzed by flow cytometry 7 days later. (B) An aliquot of cells shown in A was analyzed by immunoblotting using the indicated antibodies. (TIFF) [file ppat.1006950.s010.tiff]
